# Initial Primer Synthesis of a DNA Primase Monitored by Real-Time NMR Spectroscopy

**DOI:** 10.1021/jacs.3c11836

**Published:** 2024-03-27

**Authors:** Pengzhi Wu, Johannes Zehnder, Nina Schröder, Pascal E. W. Blümmel, Loïc Salmon, Fred. F. Damberger, Georg Lipps, Frédéric H.-T. Allain, Thomas Wiegand

**Affiliations:** † Department of Biology, Institute of Biochemistry, ETH Zürich, 8093 Zurich, Switzerland; ‡ Laboratory of Physical Chemistry, ETH Zürich, 8093 Zurich, Switzerland; § Institute of Technical and Macromolecular Chemistry, RWTH Aachen University, Worringerweg 2, 52074 Aachen, Germany; ∥ Institute of Chemistry and Bioanalytics, University of Applied Sciences Northwestern Switzerland, Hofackerstrasses 30, 4132 Muttenz, Switzerland; ⊥ Max-Planck-Institute for Chemical Energy Conversion, Stiftstr. 34-36, 45470 Mülheim an der Ruhr, Germany

## Abstract

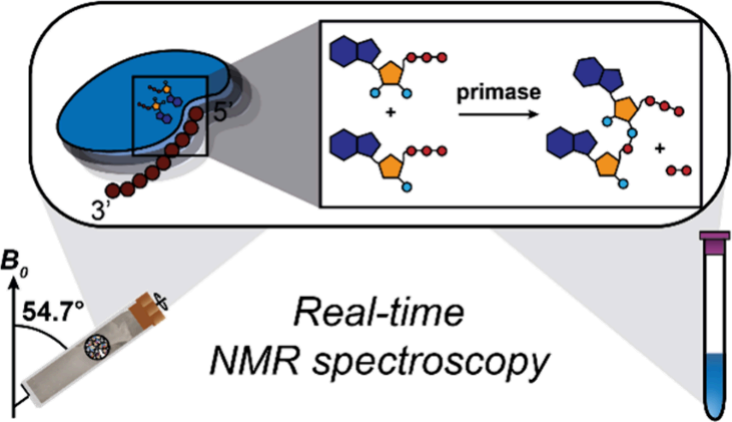

Primases are crucial enzymes for DNA replication, as they synthesize a short primer required for initiating DNA replication. We herein present time-resolved nuclear magnetic resonance (NMR) spectroscopy in solution and in the solid state to study the initial dinucleotide formation reaction of archaeal pRN1 primase. Our findings show that the helix-bundle domain (HBD) of pRN1 primase prepares the two substrates and then hands them over to the catalytic domain to initiate the reaction. By using nucleotide triphosphate analogues, the reaction is substantially slowed down, allowing us to study the initial dinucleotide formation in real time. We show that the sedimented protein–DNA complex remains active in the solid-state NMR rotor and that time-resolved ^31^P-detected cross-polarization experiments allow monitoring the kinetics of dinucleotide formation. The kinetics in the sedimented protein sample are comparable to those determined by solution-state NMR. Protein conformational changes during primer synthesis are observed in time-resolved ^1^H-detected experiments at fast magic-angle spinning frequencies (100 kHz). A significant number of spectral changes cluster in the HBD pointing to the importance of the HBD for positioning the nucleotides and the dinucleotide.

## Introduction

DNA primases are essential enzymes in DNA replication that synthesize short RNA primers, which are about 4–15 nucleotides in length, and are used by DNA polymerases to begin replication.^1^ The primases synthesize a single RNA primer on the leading strand and repeatedly on the lagging strand to facilitate the synthesis of Okazaki fragments.^2,3^ Subsequently, during Okazaki fragment maturation, the RNA primer is removed, the resulting gap is filled by DNA polymerase I, and DNA ligase seals the nick to create a continuous DNA strand.^4^ Replicative cellular DNA primases belong to two distinct classes: The DnaG and the archaeo-eukaryotic primases (AEP).^1^ Despite their functional similarity, they differ in structure and evolved independently. DnaG primases, primarily found in bacteria and bacteriophages, are associated with replicative DNA helicases and consist of three functional domains: An N-terminal zinc-binding domain involved in recognizing sequence-specific DNA, a middle RNA polymerase domain, and a C-terminal domain that either acts as a DNA helicase or interacts with a DNA helicase.^5^ The archaeo-eukaryotic primases are predominantly found in archaea and eukaryotes. They typically form a heterodimeric complex containing two subunits of approximately 49 and 59 kDa,^6^ with the smaller subunit (PriS) containing the active site and requiring the assistance of the large subunit (PriL) to synthesize a primer. In eukaryotes, this heterodimer forms a complex with the DNA polymerase α subunits (p180 and p70) that together initiate DNA replication.^6^

Archaea have homologues of eukaryotic PriS and PriL, but do not require subunits of polymerase α for primase activity.^7^ Another difference of archaea compared to eukaryotes is that DNA primases not only exist as a heterodimer but also can occasionally be found as a trimer or as a single protein containing two separate domains. For example, PriS and PriL in *Saccharolobus solfataricus* form a complex with a third small subunit, PriX.^8,9^ In contrast, the plasmid pRN1 primase in *Sulfolobus islandicus* and the primase from *Nanoarchaeum equitans*(10) are monomeric primases encompassing the domains homologous to PriS and PriL. PriX in *Saccharolobus solfataricus* and the C-terminal domain of pRN1 primase in *Sulfolobus islandicus* as well as the C-terminal domain of the *Nanoarchaeum equitans* primase fold into a helix-bundle domain (HBD), which is a structural orthologue of the C-terminal domain of the eukaryotic PriL.^11,12^ The HBD of all these proteins have been suggested to be the binding site for the initiating nucleotides during primer synthesis, and deletion of these domains abrogates primase initiation but not elongation.^8,13,14^ However, the exact mechanism by which DNA primases transfer these initiating nucleotides from the HBD to the active site, which is located in PriS, is still unknown.

Primases, such as the DnaG primase from *E. coli*, synthesize RNA primers at a rate of one primer per second.^15^ For archaeo-eukaryotic primases like pRN1, the rate is similar (10 primers per min).^16^ The primer synthesis consists of three distinct steps: Initiation, elongation, and termination. The initiation, which is the formation of the first dinucleotide, involves the binding of the first two nucleotide triphosphates (NTPs) and is often the rate limiting step.^17^ Although the PriS subunit of the human primase in isolation appears capable of initiating primer synthesis, it is much less efficient and highly unstable compared to the PriS-PriL complex.^18,19^ Therefore, PriL should play an important role in the substrate preparation for dinucleotide formation, which involves the binding of the first two cognate NTPs and subsequently facilitates their base pairing with the single-stranded DNA template. However, the reaction mechanism is largely unclear.

NMR spectroscopy is an ideal tool for studying chemical and biochemical reactions in real time benefiting from the ease to distinguish different entities based on the NMR chemical-shift values, as well as from the intrinsically quantitative nature of NMR. This allows measurement of reaction kinetics by both, solution- and solid-state NMR.^20^ Chemical reactions occurring in the magic-angle spinning (MAS) NMR rotor and followed by real-time NMR have been reported, for instance for battery materials (for a review see ref (21)), or for mechanochemical transformations (for selected examples see refs (22−26)). Biochemical reactions have also been studied in real time in the MAS rotor, such as for instance amyloid-β self-assembly processes,^27,28^ the enzymatic degradation of polyethylene terephthalate,^29^ drug binding,^30^ cellular processes^31^ or structural transitions of silk proteins.^32^ Solid-state NMR on proteins is nowadays often performed on sedimented protein samples or complexes thereof, which are typically prepared by directly sedimenting the protein in the MAS rotor in an external ultracentrifuge.^33−36^ Under these conditions, around 50% of the NMR rotor is filled with protein, whereas the rest contains buffer solution (the “supernatant”).^35,36^ This has enabled the investigation of ATP hydrolysis in real-time inside the NMR rotor.^37,38^ For instance, the hydrolysis of ATP in the ATP-fueled ABC transporter MsbA was followed by real-time ^31^P experiments detecting the nucleotides in the supernatant of the NMR rotor.^39^ A similar approach was applied for studying ATP hydrolysis and diacylglycerol phosphorylation in the membrane protein diacylglycerol kinase.^40^ Quite recently, also light-induced biological reactions were studied by real-time NMR in the solid-state NMR rotor employing the uncaging of photolabile protecting groups.^41^

We previously combined solution- and solid-state NMR to determine the structure of a quaternary complex of the HBD domain of *Sulfolobus islandicus* pRN1 primase bound to its DNA template and two ATP molecules.^13^ The structure revealed that the binding of the two ATP molecules to the HBD allowed the sequence-specific recognition of the DNA template at a GTG triplet. This quaternary complex structure also suggested that the two NTPs bound to the HBD may serve as substrates for the initial dinucleotide synthesis, but this could not be experimentally proven since the NTPs employed (ATP in this case) were not base-pairing to the DNA template used.^13^

We herein investigate the initial dinucleotide-formation process in the primase part of the multifunctional replication protein from the archaeal plasmid pRN1^16,42−44^ by real-time NMR spectroscopy in solution as well as in the solid state, with the latter employing a sedimented protein sample.^33,34^ We show evidence that the two NTPs bound by the HBD are indeed used as substrates for dinucleotide formation catalyzed by archaeal primase. Use of NTP-analogues^45,46^ was critical to prove this, since the reaction could be slowed down sufficiently to observe it in real time by NMR in solution and in the solid state. ^31^P cross-polarization (CP) experiments allow the direct detection of immobilized (protein bound) NTP species^47^ and indeed reveal the formation of a dinucleotide and allow measurement of the catalytic rate. The protein concentration in such sediments is of similar magnitude to the one in cells.^48^ Conformational changes in the protein upon dinucleotide formation were quantified in real-time by ^1^H MAS experiments at 100 kHz MAS (for biomolecular fast MAS see exemplary refs (49−58) and for very comprehensive review articles (59) and (60)) and reveal conformational changes occurring in the HBD.

## Results

### Cognate NTPs can bind the DNA template and the HBD by displacing noncognate ATPs

**Figure 1:**
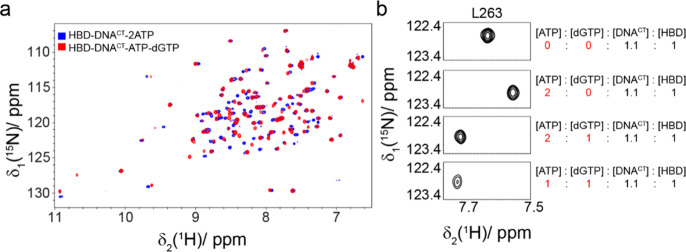
Binding of two cognate NTPs to HBD. (a) Overlay of the ^1^H–^15^N HSQC spectra of HBD-DNA^CT^-2ATP (shown in blue) and HBD-DNA^CT^-ATP-dGTP (shown in red). (b) Close-up view of CSP of L263 upon the addition to HBD-DNA^CT^ of varying ratios of ATP and dGTP. The panels from top to bottom illustrate the complex of HBD-DNA^CT^ under different conditions: without NTP, with two ATP molecules, with two ATP molecules and one dGTP molecule, and with one ATP molecule and one dGTP molecule, respectively. The data reveal that HBD binds one ATP and one dGTP, as evidenced by the shift of residue L263.

**Scheme 1:**
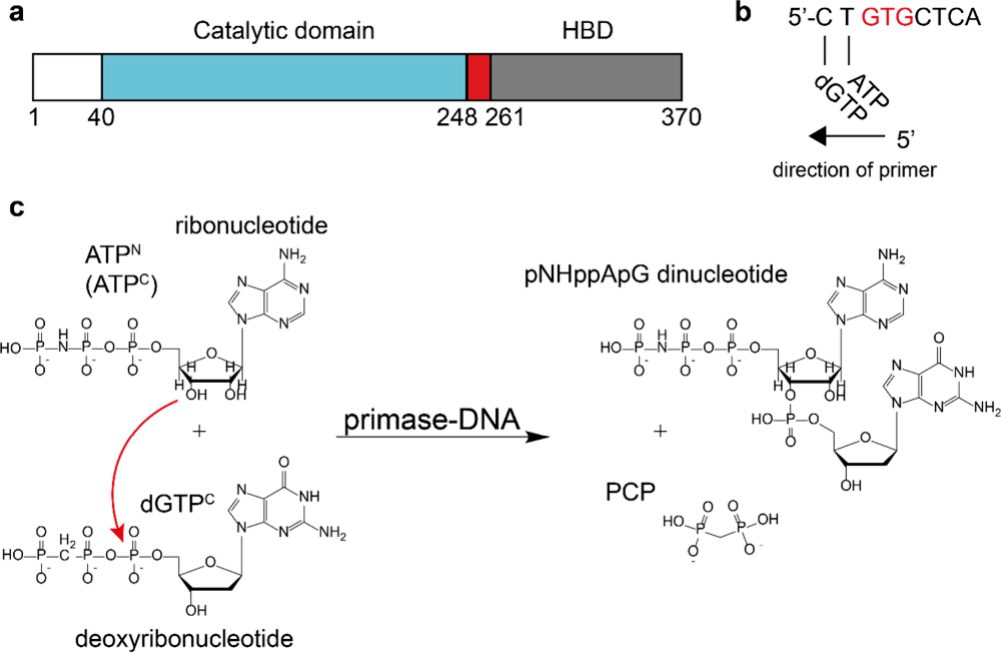
(a) Schematic Representation of the Different Domains Used in the Primase Construct; Residues 40-248 Constitute the Catalytic Domain and Residues 261-370 the Helix Bundle Domain; (b) Schematic Representation of the Base Pairing of ATP and dGTP with the DNA Template Containing the GTG Motif; (c) Schematic Drawing of the Dinucleotide Formation Catalyzed by the pRN1 Primase with NTP Analogues; the first base of the dinucleotide is exclusively a ribonucleotide, and the second one is exclusively deoxyribonucleotide

In order to investigate dinucleotide formation, we utilized protein constructs from *Sulfolobus islandicus* pRN1 containing the active primase domain (amino acids 40–370), the catalytic domain only (amino acids 40–248), or the HBD only (amino acids 256–370), which were subsequently examined using NMR spectroscopy (Scheme 1a). To study whether the two NTPs bound by the HBD may be used as a substrate for dinucleotide formation, we first investigated if the two cognate NTPs (ATP and dGTP) may bind the HBD-DNA^CT^ complex. The choice of ATP and dGTP (or their corresponding analogues) aligns with the nature of the DNA template 5′-CTGTGCTCA-3' used in our work. In this context, the first cognate rNTP (ATP) is expected to base pair with the first nucleotide upstream of the GTG motif, and the second cognate dNTP (dGTP) should base pair with the second nucleotide upstream of the GTG motif (Scheme 1b). The ^1^H–^15^N HSQC NMR spectrum of this quaternary complex showed clear spectral differences when compared with the spectrum of HBD-DNA^CT^-2ATP (Figure 1a). Moreover, titration of dGTP in a solution of HBD-DNA^CT^-2ATP led to the substitution of one ATP molecule by dGTP (Figure 1b). However, addition of the other three dNTPs (dATP, dCTP or dTTP) to the same complex does not induce chemical-shift perturbations (CSPs, Figure S1). These results suggest that dGTP binding is specific and leads to base pairing with the DNA template. Although these initial data indicate that cognate NTPs bind better to HBD-DNA^CT^, they do not prove that these NTPs are used as substrates for initial primer synthesis. Next, we therefore followed dinucleotide formation using time-resolved solution-state NMR of the active primase bound to DNA^CT^ and the cognate NTPs (ATP and dGTP). The reaction was very fast as dinucleotide formation with the cognate NTPs was terminated within several minutes. We therefore decided to use NTP analogues to slow down the catalytic activity of the enzyme in order to follow the catalytic reaction by real-time NMR spectroscopy.

### Search for NTP Analogues That Still Allow Dinucleotide Formation

**Figure 2:**
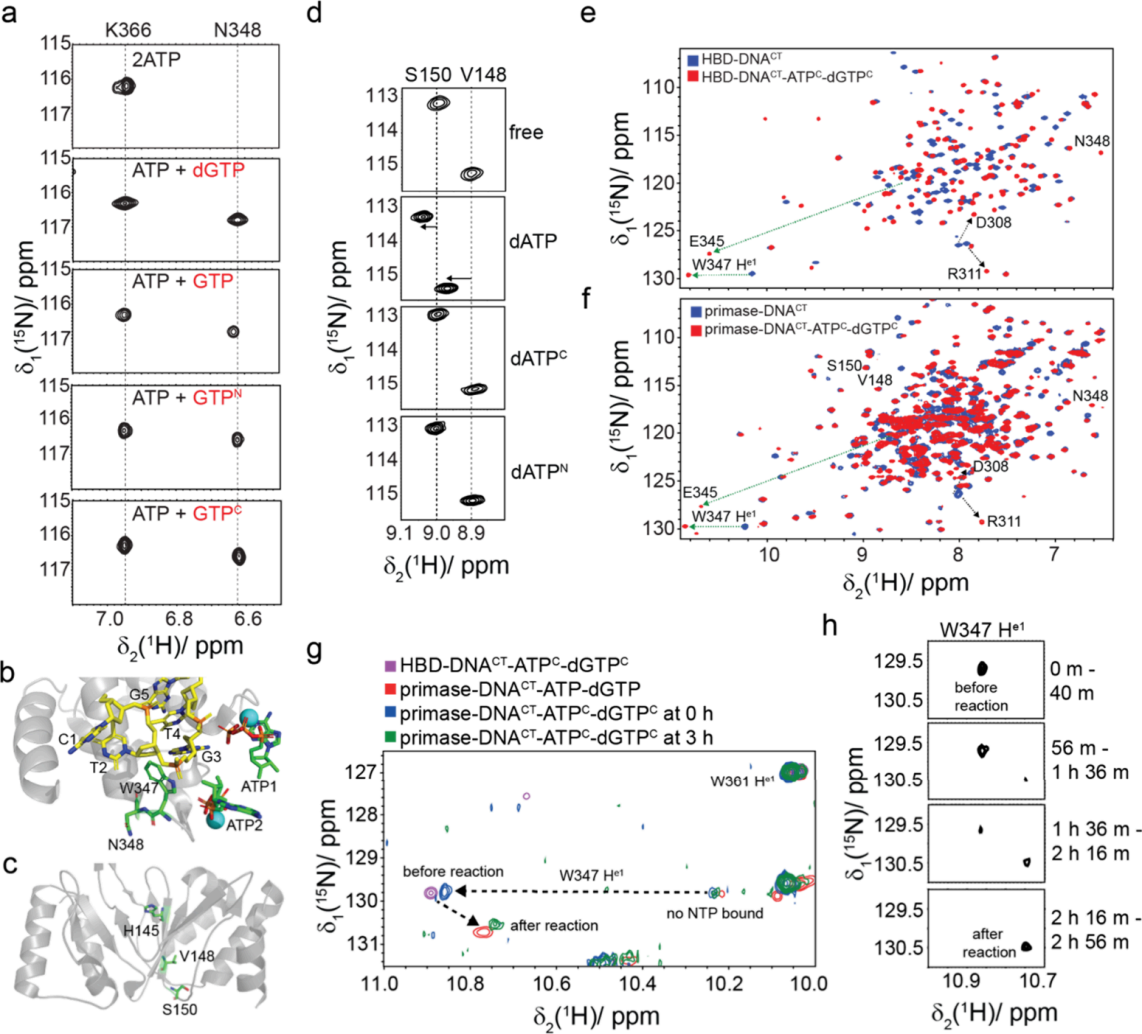
Facilitation of dinucleotide formation by NTP analogues. (a) CSP of residue N348 in complexes of HBD-DNA^CT^ with various NTP analogues. From top to bottom, the complexes are HBD-DNA^CT^-2ATP, HBD-DNA^CT^-ATP-dGTP, HBD-DNA^CT^-ATP-GTP, HBD-DNA^CT^-ATP-GTP^N^, and HBD-DNA^CT^-ATP-GTP^C^. (b) NMR structure of HBD-DNA^CT^-2ATP (PDB accession code 6GVT) showing the positions of residues W347 and N348. (c) Crystal structure of the catalytic domain (PDB accession code 1RO2) showing the positions of V148 and S150. (d) Chemical-shift changes of residues V148 and S150 in complexes of the catalytic domain with various NTP analogues. From top to bottom, the complexes are the apo catalytic domain, catalytic domain-dATP, catalytic domain-dATP^C^, and catalytic domain-dATP^N^. (e) Overlay of 2D ^1^H–^15^N HSQC spectra of HBD-DNA^CT^ and (f) primase-DNA^CT^ in the absence (blue) or presence (red) of two NTP analogues (ATP^C^ and dGTP^C^). (g) W347 ^1^H^ε1^–^15^N^ε1^ correlations in 2D ^1^H–^15^N HSQC spectra of HBD-DNA^CT^-ATP-dGTP (purple, mimicking the state before the reaction occurs, 298 K), primase-DNA^CT^-ATP-dGTP (red, representing a state after the reaction, 313 K), primase-DNA^CT^-ATP^C^-dGTP^C^ shortly after incubation (blue, mimicking the state at the beginning of the reaction, 313 K) and primase-DNA^CT^-ATP^C^-dGTP^C^ 3 h after incubation (green, mimicking a state after the reaction, 313 K). For comparison, also the W347 ^1^H^ε1^-^15^N^ε1^ correlation peak for a state without any NTPs bound is indicated. (h) W347 ^1^H^ε1^–^15^N^ε1^ correlation peaks in 2D ^1^H–^15^N HSQC spectra of primase-DNA^CT^-ATP^C^-dGTP^C^ recorded at different time points after incubation, revealing spectral changes associated with dinucleotide formation.

We screened various NTP analogues by titrating them into the HBD-DNA^CT^-2ATP complex (Figures 2a and S2). While GTP is not a substrate for primase activity, we found that it could bind to the HBD domain in the same manner as for the dGTP substrate. NMR titration results revealed that only GTP analogues with an oxygen atom substitution between the Pβ and Pγ of GTP were able to bind the HBD domain (namely GppCH_2_p and GppNHp, referred to subsequently as GTP^C^ and GTP^N^, respectively), whereas GTP analogues with substitutions at other positions could not replace ATP (namely GTPαS, GpCpp, and GTPγS). Binding of the GTP analogues containing a CH_2_ or a NH substitution for an oxygen between Pβ and Pγ resulted in similar spectra to binding of GTP or the active dGTP as exemplified by the peak position of N348 (Figure 2a). N348 is located in loop K340–N348 that weakly binds the second NTP and is absent in the NMR spectra of HBD-DNA^CT^ bound to ATP due to conformational exchange (Figure 2b). The N348 resonance is visible in the spectra of HBD-DNA^CT^ bound to dGTP, GTP, GTP^C^ and GTP^N^ due to increased binding affinity and rigidification of loop K340–N348.

We also titrated two different dATP analogues into the free catalytic domain to study whether the catalytic domain could also bind to dNTP analogues. The resonances of V148 and S150, which are located in close proximity to the active site, were examined (Figure 2c). Our findings indicate that the addition of dATP to the catalytic domain resulted in visible CSPs of V148 and S150. The amide protons of V148 and S150 experience proton chemical-shift changes between 0.05 and 0.1 ppm upon dATP binding. In contrast, dATP^C^ and dATP^N^ failed to induce any changes (Figure 2d). Therefore, it appears that in contrast to the HBD, the catalytic domain is apparently unable to bind any of the tested dATP analogues.

Next, it was probed whether the construct containing the active primase (containing the HBD and the catalytic domain) would bind and possibly react with such NTP analogues. To investigate this, both ATP^C^ and dGTP^C^ were rapidly added to the HBD-DNA^CT^ complex (Figure 2e) or the primase-DNA^CT^ complex (Figure 2f), and then 2D ^1^H–^15^N HSQC spectra were immediately collected. We found that initially only residues in the HBD domain showed chemical-shift changes, while the catalytic domain did not. These results indicate that at the beginning of this experiment, the two NTP analogues bind to the HBD domain but not to the catalytic domain. After monitoring the reaction with ATP^N^-dGTP^C^ or ATP^C^-dGTP^C^ for a few hours, we could observe evidence of dinucleotide formation at a very slow rate (Figure 2g). Indeed, in Figure 2g when focusing on the W347 ^1^H^ε1^–^15^N^ε1^ correlation upon NTP binding (which serves as a sensitive probe to follow both conformational changes in the HBD and dinucleotide formation), we see initially large CSPs indicating binding to the HBD and after 3 h a shift indicative of dinucleotide formation, as the spectrum is almost identical to the one obtained after the reaction with unmodified NTPs.

Taking together, these results provide evidence that during the dinucleotide formation, the HBD domain itself prepares both substrates, which are the first two cognate NTPs, while the catalytic domain does not participate in this process. Once the HBD domain has prepared both substrates, it can interact with the catalytic domain, delivering them into the active site of the catalytic domain to initiate the reaction. A slow exchange time course of the reaction can be followed when using the NTP analogues (Figure 2h).

### Initial Time-Resolved ^1^H–^15^N HSQC NMR Studies of Dinucleotide Formation Using NTP Analogues

We next investigated using real-time solution- and solid-state NMR spectroscopy the dinucleotide formation reaction in the primase (residues 40–370), which comprises the HBD and catalytic domain with the nucleotide analogues. After binding of ATP^N^ (or ATP^C^) and dGTP^C^, the enzyme catalyzes the formation of a dinucleotide (referred to as pNHppApG or pCppApG dinucleotide, respectively) as the initial primer for DNA replication, during which a bisphosphonate (PCP) is released (Scheme 1c). To investigate this process, primase pRN1 is incubated with single-stranded DNA^CT^ and cognate nucleotides ATP^N^ and dGTP^C^ to initiate the reaction. Dinucleotide formation can be nearly entirely suppressed by using the catalytically inactive mutant H145A that serves as a control in our studies.^61^

Initial time-resolved ^1^H–^15^N HSQC spectra of primase-DNA^CT^-ATP^C^-dGTP^C^ allowed us to monitor the dinucleotide formation reaction in real-time within the NMR tube (Figure 2h). The possible origin for the much slower reaction kinetics might be that the NTP analogues are not easily handed over to the catalytic domain, as this domain in isolation cannot bind these modified NTPs (Figure 2d).

### ^31^P Resonance Assignment of Unbound and Bound Nucleotides

After establishing that NTP analogues bind to the protein and slow down the dinucleotide formation reaction substantially, we employed NMR spectroscopy in solution as well as in the solid state to explore whether real-time phosphorus-31 NMR can be used in both aggregate states to follow the reaction and to obtain kinetic information. We showed previously that pRN1 is highly suitable for solid-state NMR studies, since it forms a highly concentrated sediment in the MAS NMR rotor.^13^ Sedimented pRN1 protein samples^33−35^ were obtained after ultracentrifugation overnight in the presence of 1eq DNA^CT^ and 2eq ATP^C^ as well as 2eq dGTP^C^. It is important to note that the ultracentrifugation time obviously remains a blind spot for our real-time approach.

**Figure 3:**
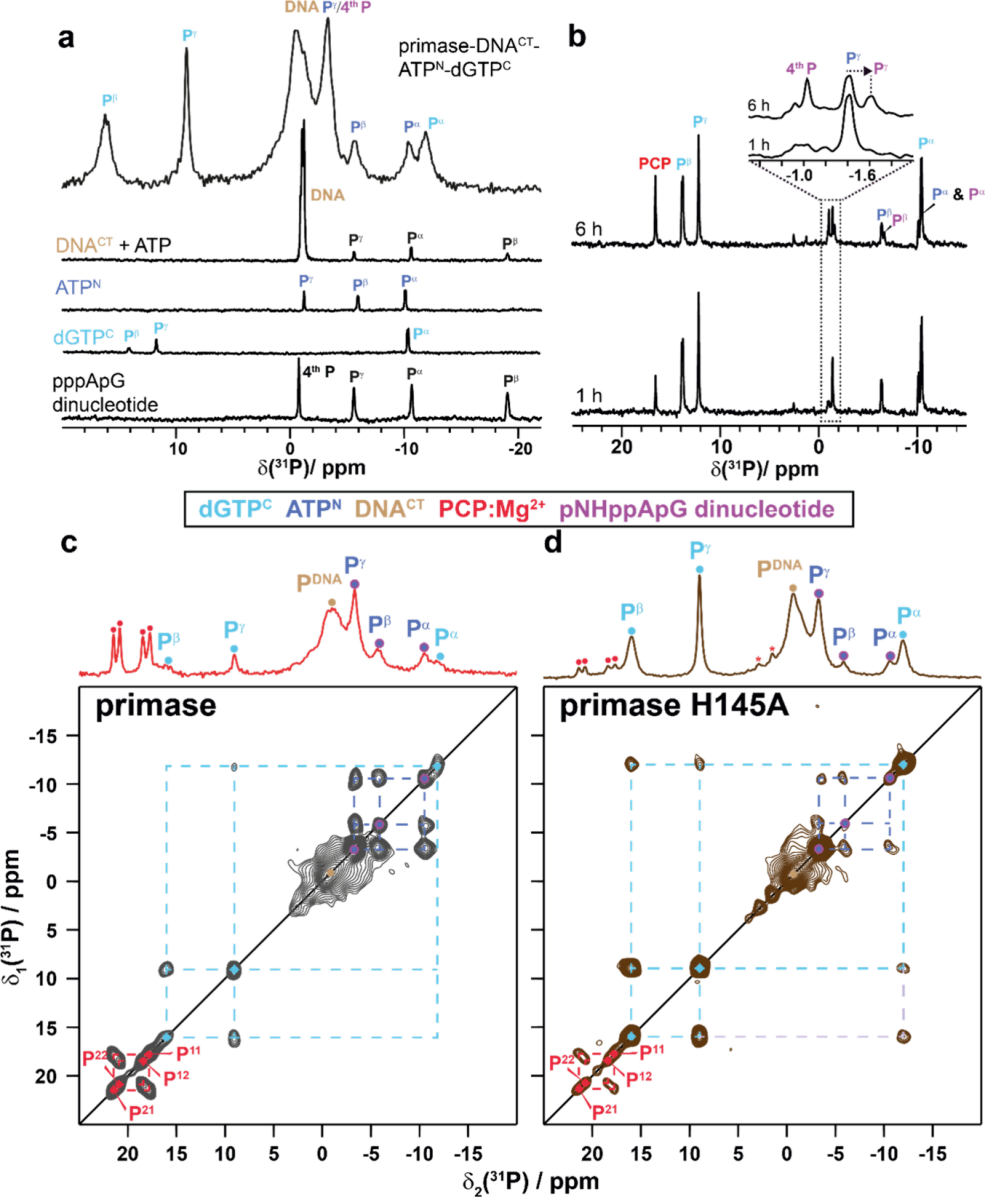
^31^P solid-state NMR spectrum and solution-state NMR spectra of free nucleotides allow for resonance assignment of bound nucleotides. (a) ^31^P solution-state NMR spectra of DNA and ATP, ATP^N^, dGTP^C^, the wild-type pppApG dinucleotide similar to the one formed by the primase-catalyzed reaction and the ^31^P CP-MAS spectrum of primase-DNA^CT^-ATP^N^-dGTP^C^ (recorded at 11.7 T and an MAS frequency of 17 kHz) at 281 K. In all cases, the same Mg^2+^ concentrations and buffer as for the samples with protein have been used. (b) ^31^P solution state NMR spectra of the complex primase-DNA^CT^-ATP^N^-dGTP^C^ with a ratio of 1:1.1:50:50 recorded 1 and 6 h after complex formation at 323 K. The zoom highlights the phosphodiester ^31^P resonance at −1.0 ppm (referred to as the 4th P) of the formed pNHppApG dinucleotide. Notably, the P^γ^ signal of free ATP^N^ experiences a chemical-shift change from −1.4 to −1.6 ppm upon dinucleotide formation. (c) 2D ^31^P–^31^P solid-state NMR 150 ms DARR spectra of primase-DNA^CT^-ATP^N^-dGTP^C^ and (d) primase-H145A-DNA^CT^-ATP^N^-dGTP^C^. Both spectra were recorded on samples obtained 16 h after the rotor filling in the ultracentrifuge. The 1D spectra with the corresponding resonance assignments are shown on top of the 2D spectra. The spectra were recorded at 11.7 T and 17 kHz MAS.

Figure S3 shows the ^1^H–^31^P solid-state CP MAS spectrum of primase-DNA^CT^-ATP^C^-dGTP^C^ in which only the immobilized (protein-bound) nucleotides and nucleic acids are visible. The ^31^P resonance assignment is deduced from the cross-peak pattern in a 2D ^31^P–^31^P Dipolar Assisted Rotational Resonance (DARR)^62,63^ spectrum (Figure S4) indeed showing the bound dGTP^C^ and ATP^C^ as well as the DNA. This is further confirmed by the ^31^P solution-state NMR spectra recorded on control solutions of NTPs, NTP analogues and DNA only (Figure 3a). The fact that most of the ^31^P solid-state NMR resonances of dGTP^C^ and ATP^C^ overlap (except for Pα for which two separate resonances are observed) would severely complicate the kinetic studies if performed with this sample.

We thus replaced ATP^C^ by ATP^N^ allowing a clear distinction of the ^31^P resonances of ATP^N^ from those of dGTP^C^ (Figure 3a). We performed ^31^P solution-state NMR experiments with a large excess of NTPs (both ATP^N^ and dGTP^C^) with respect to the DNA template (50 times the molar ratio) at 323 K to directly observe the dinucleotide signal. These spectra allow an unambiguous identification of the phosphodiester ^31^P resonance of the formed dinucleotide in solution at around −1 ppm which builds up over time (Figures 3b and S5). Additionally, at this increased temperature, we successfully achieved separation between the triphosphate signals of ATP^N^ and the dinucleotide. To obtain kinetic information, we then adjusted the NTPs/DNA ratio from 50 to 2 and lowered the temperature from 323 to 281 K. Figure 3c shows the ^31^P–^31^P DARR solid-state NMR spectrum recorded on primase-DNA^CT^-ATP^N^-dGTP^C^ 16 h after rotor filling enabling the resonance assignment, again complemented by the solution-state NMR spectra of the free nucleotides (Figure 3a). Figure 3d displays the ^31^P–^31^P DARR spectrum of the catalytically inactive mutant H145A. Interestingly, in both spectra (Figure 3c-d) four sharp resonances at ^31^P chemical-shift values of 17.7, 18.5, 20.8, and 21.5 ppm appear, which are more intense in the spectrum of the wild-type (wt) protein. These resonances were assigned based on the 2D DARR spectrum to the bisphosphonate species (PCP) that is the second product of dinucleotide formation (Scheme 1c) and possibly precipitates as Mg^2+^:PCP with two crystallographically distinct PCP molecules in the asymmetric unit, as for instance observed in crystalline zoledronate (see also Figures S6–S8).^64^ The ability of precipitation has also been observed for Mg^2+^:pyrophosphate formed in the context of nucleic acid amplification.^65,66^

### Dinucleotide Formation Followed by Real-Time ^31^P NMR

**Figure 4:**
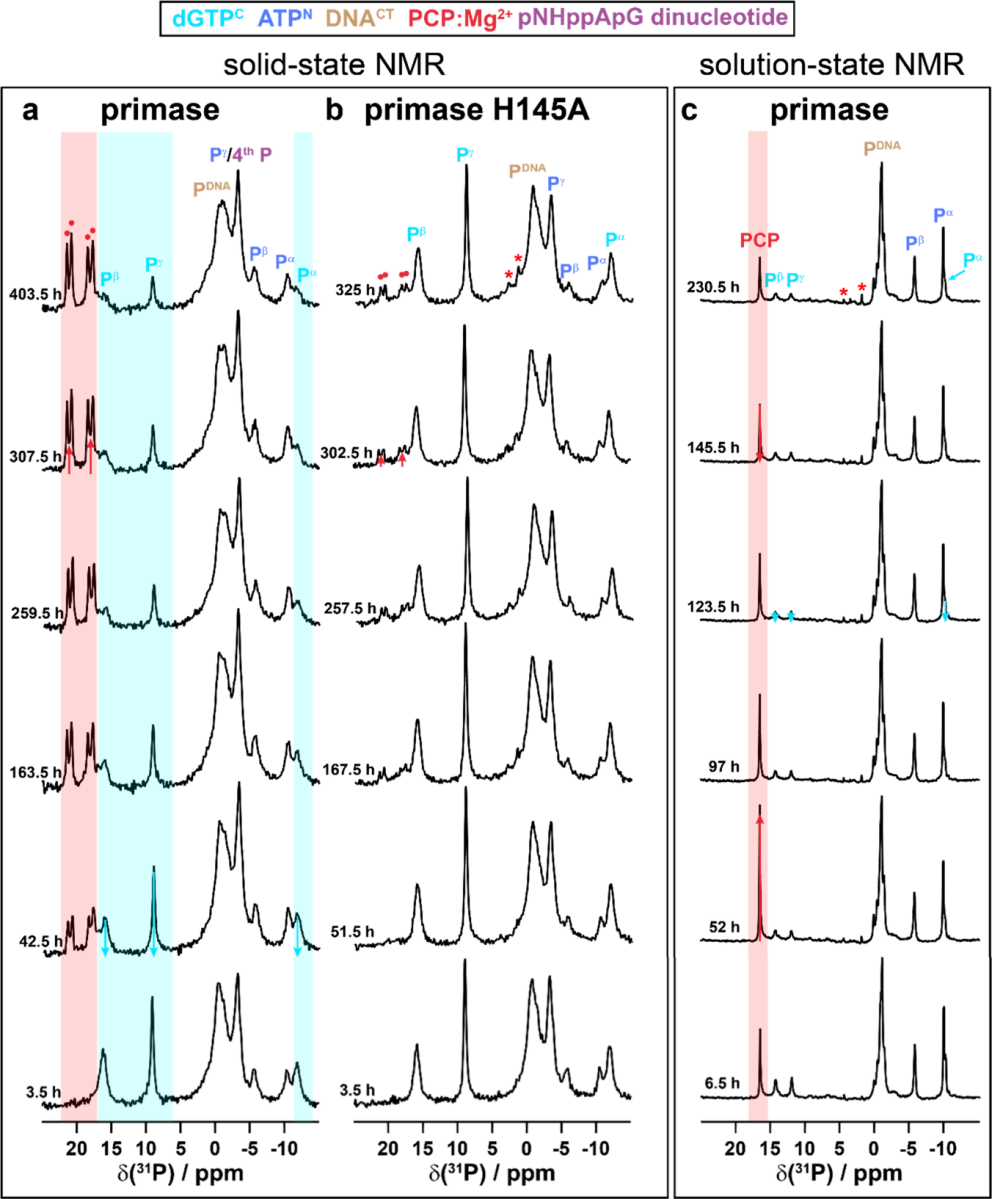
Time-resolved ^31^P NMR spectra enable to follow the pRN1 primase-catalyzed dinucleotide formation occurring in the solid state and in solution. (a) ^1^H–^31^P CP-MAS of primase-DNA^CT^-ATP^N^-dGTP^C^, (b) primase-H145A-DNA^CT^-ATP^N^-dGTP^C^, and (c) solution-state ^1^H-decoupled ^31^P spectra of primase-DNA^CT^-ATP^N^-dGTP^C^. Colored boxes indicate the most significant spectral changes occurring over time. Resonances marked by an asterisk are assigned tentatively to hydrolysis products of ATP^N^ (AMP and P_i_).

We next turned to time-dependent ^31^P NMR experiments in solution and in the solid state. Figure 4 shows the time-dependent ^1^H–^31^P solid-state CP-MAS spectra of wt primase and the mutant H145A bound to DNA^CT^, ATP^N^ and dGTP^C^. The spectra recorded 3.5 h after spinning-up the rotor are highly similar for both cases and reveal ^31^P resonances of bound DNA (colored in brown in Figure 4) and three resonances for each triphosphate (ATP^N^ colored in blue and dGTP^C^ in cyan). Over time, the dGTP^C^ resonances decrease and the PCP resonances increase for the wt protein. The constant ^31^P resonances of ATP^N^ (with a small initial build-up of the Pγ resonance overlapping with the DNA resonances, *vide infra*) point to dinucleotide formation and subsequent binding of it. The phosphodiester group of the bound dinucleotide thus resonates at a comparable ^31^P chemical-shift value to the ATP^N^ Pγ resonance (at around −3 ppm) as also confirmed by solution-state NMR in which the free dinucleotide is detected at around −1 ppm (Figure 3a). The dinucleotide still comprises the intact triphosphate unit of ATP^N^ (see Scheme 1), which remains bound to the protein. Altogether, our observations imply that we can indeed use in-situ solid-state NMR to follow the dinucleotide formation by the pRN1 primase. As a control, Figure 4b shows the time-dependent ^31^P CP-MAS spectra of the mutant H145A. And indeed, the dGTP^C^ resonances remain constant and only a slight decrease of the ATP^N^ resonances is observed, possibly indicating autohydrolysis of ATP^N^. Yet, a much weaker build-up of PCP resonances is observed as well, pointing to still a small amount of primase activity. Note, that in the wt protein also the DNA resonances decrease over time. Our NMR data together with the NMR structure of HBD-DNA-2ATP show that at the beginning of the reaction DNA and the two NTPs are initially bound by the HBD and subsequently handed over to the catalytic domain. This process implies that DNA undergoes conformational as well as dynamic changes during the reaction, resulting in different chemical shifts as well as changes in peak intensities, as experimentally observed.

Our findings are further supported by real-time ^31^P solution-state NMR experiments (see Figure 4c). Note that the protein concentrations differ significantly between the solid-state and solution-state NMR samples (around 450 mg/mL in the sedimented protein^67^ and 20 mg/mL in solution). Another difference between solution-state and solid-state NMR is that in the former, there is still 1 equiv of free ATP^N^ and free dGTP^C^ present in the NMR tube, whereas in the latter, there is much less free NTP in the rotor after the sedimentation. In contrast to the solid-state NMR CP spectra, the solution-state spectra are dominated by the unbound nucleotides which appear as sharp resonances, whereas resonances of bound nucleotides are significantly broadened. The dGTP^C^ resonances decrease over time, whereas the PCP resonances initially increase (in contrast to the solid-state NMR spectra, only a single resonance at 16.5 ppm is detected, pointing to chemical equivalence of the two phosphorus nuclei in solution). However, after several hours the PCP resonance in solution decreases as well, in agreement with a solid precipitate formed in the NMR tube (*vide infra*).

These ^31^P solid-state and solution-state time-resolved experiments further confirm that the two modified NTPs that are initially bound to the HBD are used as substrates for dinucleotide formation. It remains an important question to unravel how the NTPs can be brought from the HBD to the catalytic domain of the primase.

### Kinetic Analysis of Dinucleotide Formation in a Sedimented Sample and in Solution

**Figure 5:**
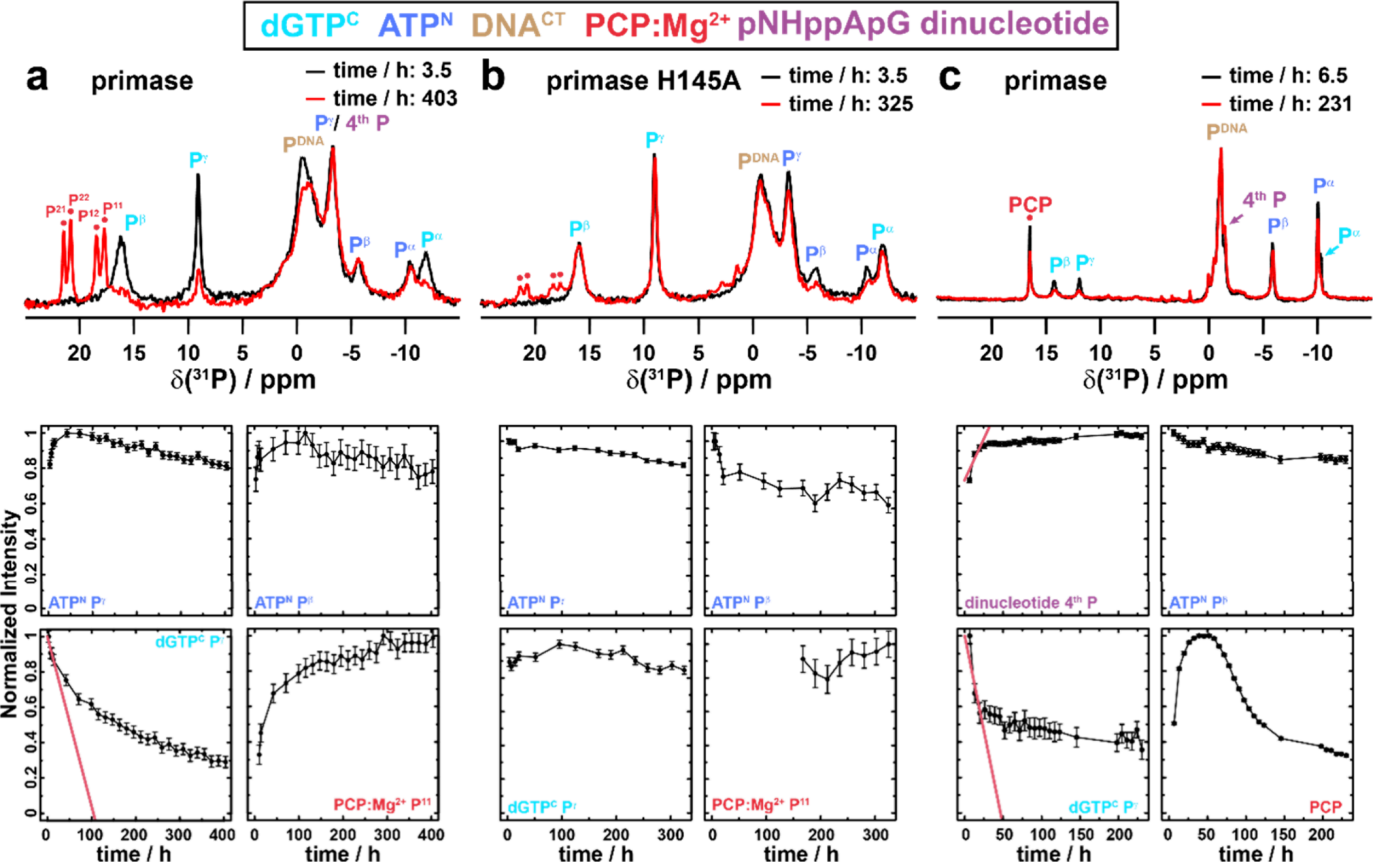
Kinetic information of dinucleotide formation obtained from a sedimented and dissolved pRN1 sample. Intensity changes as a function of time determined from the ^1^H–^31^P CPMAS spectra of primase-DNA^CT^-ATP^N^-dGTP^C^ (a), from the ^1^H–^31^P CPMAS spectra of primase-H145A-DNA^CT^-ATP^N^-dGTP^C^ (b) and from solution-state ^31^P spectra of primase-DNA^CT^-ATP^N^-dGTP^C^ (c). For further panels, see Figure S11.

We next tried to access the kinetics of the dinucleotide formation occurring in a solid-state reaction in a sedimented primase sample as well as in the NMR tube in solution. Figure 5a and b thus summarize the intensity changes determined for the wt protein and the H145A mutant in the solid-state NMR spectra. It is important to note that this analysis neglects the initial 16 h of ultracentrifugation, in which the sedimentation in the NMR rotor takes place. Furthermore, all data points have been normalized with their most intense signal to one, for instance, assuming that only negligible dinucleotide formation occurred during rotor filling and that a complete conversion has occurred in the time frame studied (∼400 h). While the first approximation seems to be decently justified, since no PCP resonances are observed in the first ^31^P CP-MAS spectrum after rotor filling (Figure 4a), the latter approximation is very crude, since no full turnover is achieved (*vide infra*). The rate constant was determined by a linear fit to the first three data points of the spectrally isolated dGTP^C^ Pγ resonance (initial reaction rate constant, red line in Figure 5), since the detailed kinetic model of the reaction is unknown. Similarly, the ^31^P solution-state NMR spectra have been quantified. An important difference in this analysis is that in the solid-state NMR spectra the decrease of the bound dGTP^C^ is quantified, whereas in solution the decrease of the free (the excess) of dGTP^C^ is followed over time. The solid-state NMR analysis thus enables a more direct observation of the enzymatic reaction. The isolated resonances of bound dGTP^C^ decrease in the solid state with an initial rate constant of 0.23 ± 0.03 d^–1^ (determined for the spectrally isolated P^γ^ resonance) compared to 0.51 ± 0.10 d^–1^ in solution (Figure 5c). We have also analyzed the build-up of the PCP resonances in the solid-state NMR spectra over time taking the incomplete conversion in the time frame studied into account. A similar rate constant for the decrease of the dGTP^C^ resonance (0.28 ± 0.09 d^–1^) has been found (for an extended discussion see Figure S9). Note that the PCP resonances can be observed in the solid-state NMR spectra due to a precipitation process, which follows the release of PCP during the dinucleotide formation. This precipitation process also leads to the multiphasic shape of the PCP intensity changes over time in the solution-state NMR spectra. The amount of detected PCP increases initially due to dinucleotide formation, but it decreases again due to the precipitation process. Of particular interest is the behavior of the ATP^N^ P^γ^ resonance that initially increases in the solid-state spectra, before it starts to decrease as well (Figure 4a). We attribute this initial increase to dinucleotide formation and subsequent binding to pRN1 primase. The phosphodiester group of the dinucleotide is expected to resonate at a similar chemical-shift value to that of the DNA phosphate groups. This is confirmed in the ^31^P solution-state NMR spectra of the unbound dinucleotide (see Figure 3b, in which the ^31^P resonance of the phosphodiester group is detected at around −1 ppm). The decrease of the −3.3 ppm resonance (assigned to bound DNA) in the solid-state NMR spectra over longer reaction times is explained by conformational and dynamic changes of bound DNA (the intensity of bound DNA decreases over time; see Figure S10). Figure 5b also shows the analysis for mutant H145A revealing nearly constant triphosphate peak intensities, despite a minor decrease of the ATP^N^ resonances possibly due to autohydrolysis.

### Following Protein Conformational Changes during Dinucleotide Formation by Proton-Detected Solid-State NMR

**Figure 6:**
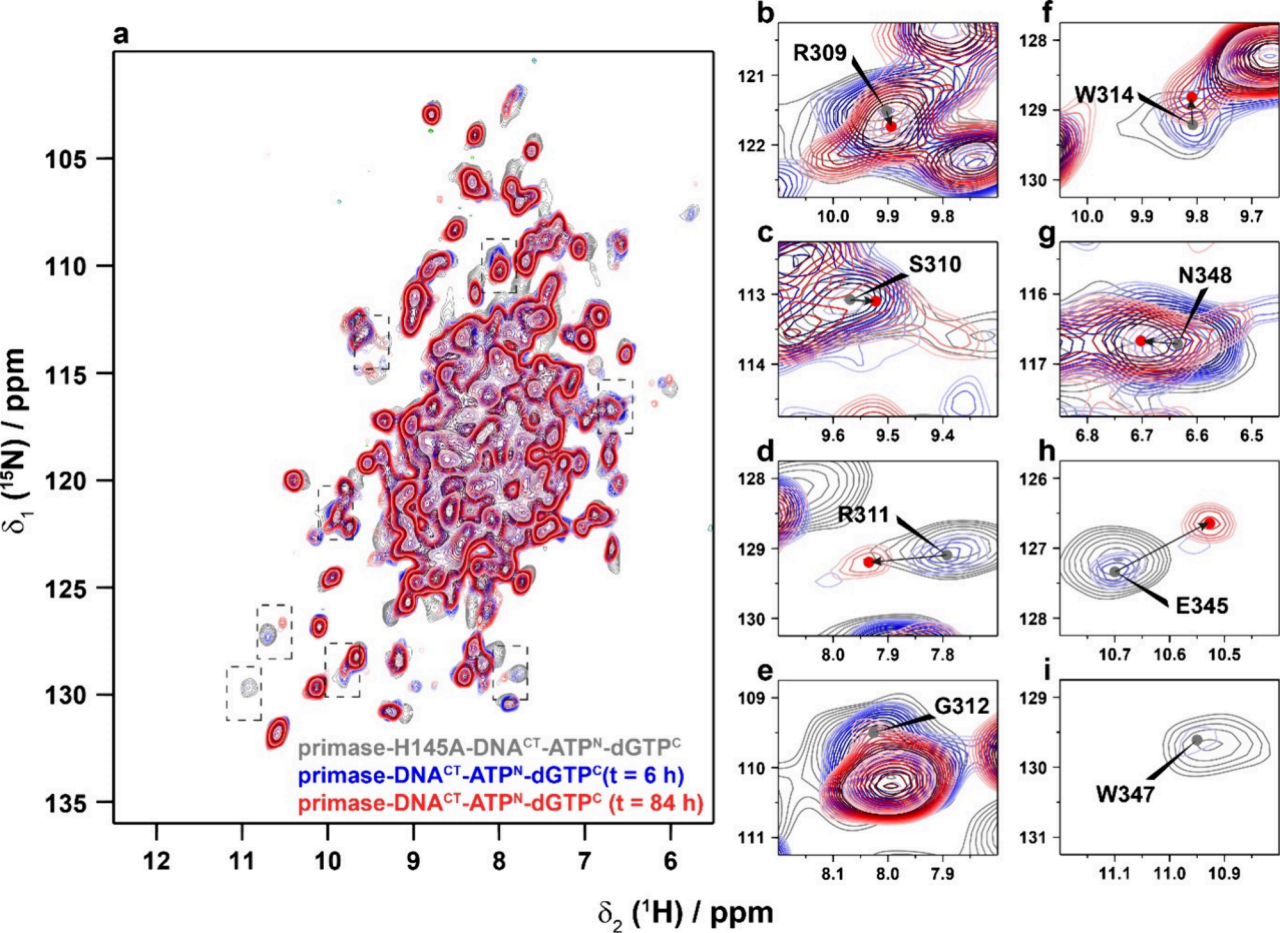
Protein conformational changes during dinucleotide formation monitored by ^1^H-detected hNH spectra at 100 kHz MAS. Time dependent 2D hNH spectra of primase-DNA^CT^-ATP^N^-dGTP^C^, as well as the control spectrum for the mutant H145A. Spectral expansions indicated in (a) are shown in (b)–(i). Note that the gray spectrum has been recorded with three times more scans compared with the real-time spectra.

Up to here, dinucleotide formation in the sedimented protein sample was investigated by detecting spectral changes of the nucleotides. However, monitoring the reaction by detecting real-time protein conformational changes would be of particular interest, as well. We have thus recorded ^1^H–^15^N hNH CP-based spectra at 100 kHz MAS. The acquisition of such spectra is possible in relatively short time (∼6 h for a reasonable signal-to-noise-ratio). Figure 6 shows two hNH spectra of primase-DNA^CT^-ATP^N^-dGTP^C^ (a deuterated and 100% back-exchanged sample was used) recorded 6 h (blue spectrum) and 84 h (red spectrum) after filling the NMR rotor. As a negative control, the hNH spectrum of mutant H145A is shown as well (gray). The spectra of the mutant mimicking the starting point of the dinucleotide formation as well as the spectrum of the wt protein shortly after sedimentation are highly similar (see also spectral zooms in Figure 6, right column). The spectrum at 84 h however reveals some spectral differences, as particularly visible for the spectrally isolated resonances R311, W314 and E345 located in the HBD (see Figure 6). For R311 and E345, at shorter reaction times, two resonances are observed in the hNH spectra, pointing to two populations of the initial ATP^N^/dGTP^C^ bound state and the final dinucleotide bound state (see Figure 6). The observation of two distinct peaks indicates a slow chemical exchange between the educt and product of the enzyme-catalyzed dinucleotide formation reaction, leading to the new appearing peaks with increasing intensity over time, whereas the signal intensities decrease over time for the resonances assigned to the protein before the reaction (*vide infra*).

**Figure 7:**
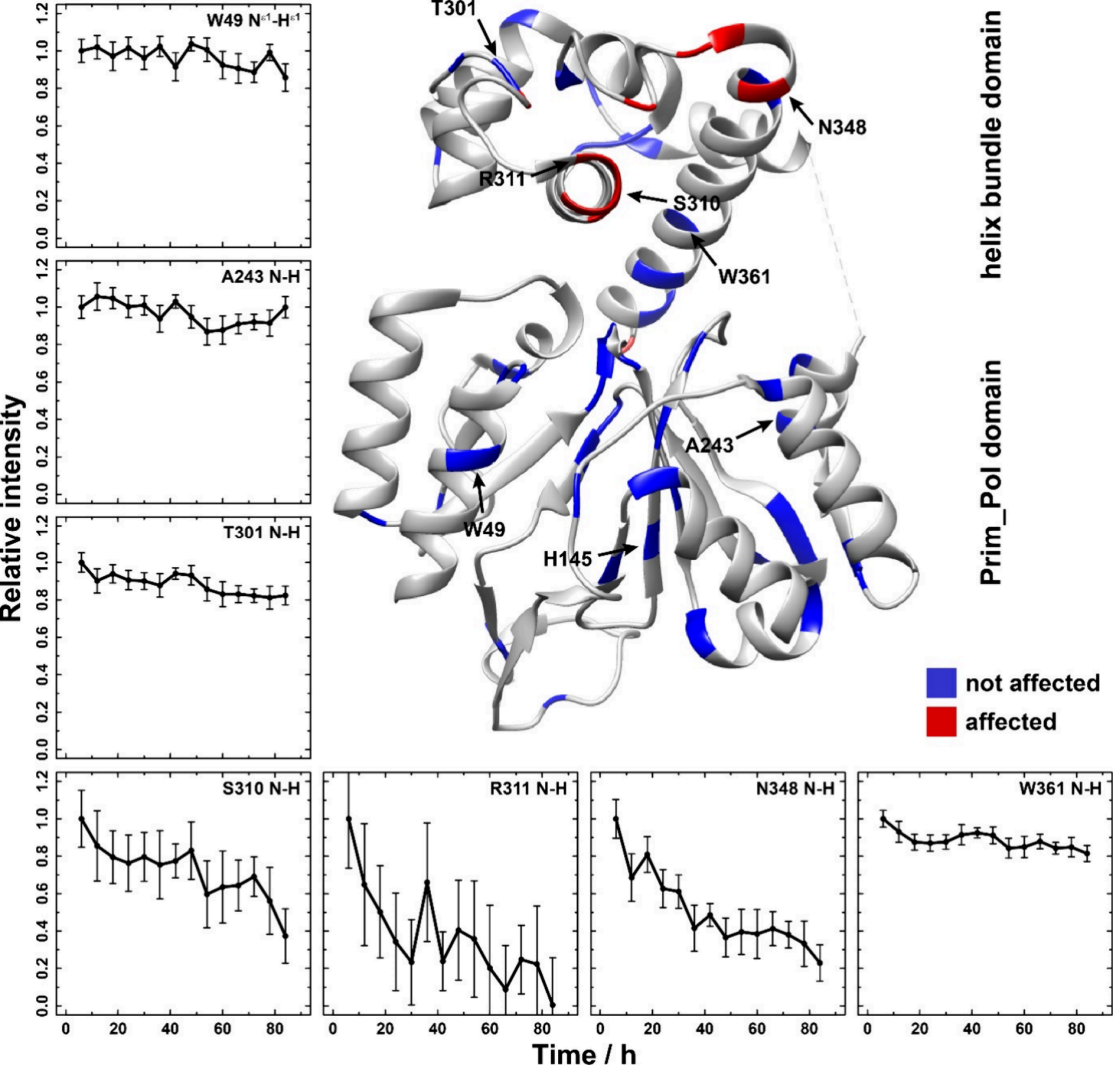
Time-resolved protein spectral changes pinpoint the dinucleotide binding site in the HBD. Representative examples of isolated peaks identified from time dependent 2D hNH spectra of primase-DNA^CT^-ATP^N^-dGTP^C^. Residues experiencing no time-dependent changes are plotted in blue and residues experiencing significant time-dependent changes are plotted in red on the crystal structure of pRN1 primase (PDB ID 3M1M).^43^

Figure 7 shows a quantification of the intensity changes in the hNH spectra, which in fact were only determined for isolated peaks in the 2D spectra. While some peaks remain constant in intensity (*e.g*., W49, A243, T301 and W361, see Figure 7), some systematically change in intensity over the course of the reaction (*e.g*., S310, R311 and N348). The latter thus experience conformational and eventually also dynamic changes during the dinucleotide formation reaction. A significant number of spectral changes cluster in the HBD thus underscoring the importance of the HBD for positioning the nucleotides and the dinucleotide. Note that the kinetics observed in the fast MAS experiments differ from the ones reported using ^31^P-detection due to a higher temperature in the MAS rotor at 100 kHz MAS.

**Figure 8:**
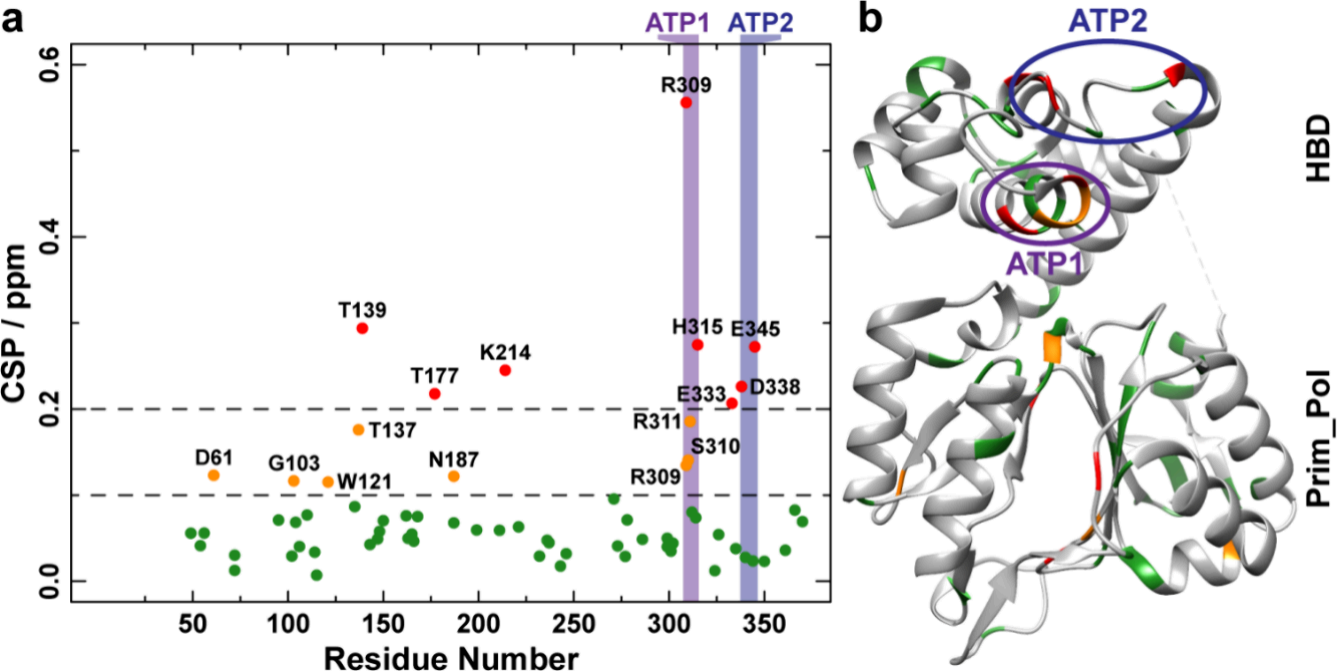
Site-specific chemical-shift perturbations between the initial state (probed by the mutant H145A due to the better signal-to-noise ratio in the spectrum compared to the wt protein) and the final state of the dinucleotide formation reaction. (a) ^1^H/^15^N CSPs determined by comparing 2D hNH spectra of primase-H145A-DNA^CT^-ATP^N^-dGTP^C^ and primase-DNA^CT^-ATP^N^-dGTP^C^ several days after rotor filling (at the end of the dinucleotide formation reaction). (b) ^1^H/^15^N CSPs (green: CSP < 0.1 ppm, orange: 0.1 ppm < CSP < 0.2 ppm, red: CSP < 0.2 ppm) plotted on the X-ray structure of primase, the nucleotide binding sites determined in previous work^13^ are marked as ATP1 and ATP2 (PDB ID 3M1M(43)).

Figure 8 and Table S1 show the CSPs between the initial state, probed by primase-H145A-DNA^CT^-ATP^N^-dGTP^C^, and the final state of the dinucleotide reaction, the latter probed by primase-DNA^CT^-ATP^N^-dGTP^C^ several days after rotor filling. Also here, significant CSPs are observed for residues within or close to the two nucleotide binding sites identified previously.^13^ However, several CSPs are also observed within the catalytic domain. It remains to be further investigated whether these changes are related to conformational changes in the catalytic domain during dinucleotide formation or to the changes caused by the H145A point mutation.

## Discussion

The present study focuses on the molecular mechanism and kinetics of primer synthesis catalyzed by a DNA primase; an essential enzyme in DNA replication that initiates the process by synthesizing short RNA primers. The rate-limiting step of primer synthesis is considered to be the dinucleotide formation. However, until now, little is known about how DNA primase prepares substrates necessary for dinucleotide formation at the molecular level. Two different mechanisms are discussed in the literature. The first one states that the catalytic domain alone is sufficient for synthesizing the dinucleotide. This mechanism is supported by a previous study demonstrating the synthesis of de novo primers in the presence of 5 mM manganese using only the human PriS subunit, representing the catalytic domain of the human primase. This suggests a less prominent role for the HBD in the dinucleotide formation.^18^ In contrast, the second mechanism implicates the HBD and the related accessory domains of DNA primases, as seen in studies such as the pRN1 primase^43^ and human primase,^14^ have a crucial function in the formation of the dinucleotide. However, the specific role of the HBD within this process has remained unclear.

In our previous NMR study,^13^ we showed that the HBD of pRN1 primase specifically recognized the “GTG” motif of the DNA template (5′-CTGTGCTCT-3′) in the presence of two ATP molecules. In the NMR structure of HBD-DNA^CT^-2ATP,^13^ it is noteworthy that, despite the spatial proximity of the two nucleotides and DNA arrangement, there is no evidence that the two ATP molecules can base pair with the DNA template. In addition, the binding of a sole triphosphate induces the same chemical-shift changes as those for ATP. These results indicate that the interactions of the two ATP molecules are not nucleotide specific. Consequently, it was only speculated, but not experimentally proven, that these two ATP molecules could represent the actual substrates for dinucleotide formation. One could not exclude that the two cognate NTPs may be bound by the catalytic domain simultaneously, which can be supported by the NTP binding capability of the free catalytic domain as shown in Figure 2d.

We herein showed that dGTP can specifically bind to the HBD-DNA^CT^-2ATP complex, providing evidence that HBD can bind the first two cognate NTPs simultaneously. We further demonstrated that these cognate NTPs are subsequently transferred to the catalytic domain, where the dinucleotide formation takes place. Our work, therefore, elucidates the critical role of the HBD in substrate preparation for dinucleotide formation that the substrates initially bound by the HBD are subsequently handed over to the catalytic domain for initiation of the dinucleotide formation reaction.

Our findings are consistent with previous work showing that the point mutations in the HBD of pRN1 primase abolish dinucleotide synthesis, but do not affect the elongation of a primer bound to DNA template.^43^ Thus HBD and the related accessory domains of other primases have a crucial function in the first step of primer synthesis, which is dinucleotide formation. For example, the crystal structure of PriL-HBD of the human primase showed that it can form a stable complex with the ssDNA template and a primer using its 5′-triphosphate for binding.^14^ The crystal structure of PriX of *Saccharolobus solfataricus*, which is also an HBD, also showed that it can bind the initiating NTP by binding to its triphosphate.^8^ As the HBD is conserved in most archaeo-eukaryotic primases, PriL of human primase and PriX of *Saccharolobus solfataricus*, which is similar to the HBD of pRN1 studied here, may also have the ability to prepare both substrates for dinucleotide formation. Although, recent results suggest that the PriS of human primase is alone capable of synthesizing primers in the presence of 5 mM manganese(II), it is much less efficient than when combined with PriL.^18^ It is worth noting that PriS alone only exhibits primase activity when the concentration of manganese(II) is higher than 100 μM, whereas the concentration of manganese(II) in cells is typically between 2 and 30 μM.^68^ Therefore, it is likely that for the human primase, the two cognate NTPs for dinucleotide formation may be handed to PriS by the PriL-HBD in human cells.

Time-resolved solid-state NMR spectra enable the investigation of the initial formation of a primer catalyzed by the archaeal primase from the plasmid pRN1, focusing either on the detection of the nucleotides by ^31^P-detected NMR or on protein conformational changes in ^1^H-detected NMR spectra at fast MAS. This is achieved by slowing down the reaction substantially using NTP-analogues. Sedimented protein samples benefit from the ease in sample preparation, since the protein is mixed with nucleotides (here two cognate NTP analogues and single-stranded DNA) prior to the ultracentrifugation process.^35,47,69^ Such sediments still remain active for enzymatic reactions (see for instance also reference^38^ for the example of the ATPase p97).

The ^31^P CP-MAS spectra reveal that dGTP^C^ hydrolyzes over time and the corresponding formation of a Mg^2+^:bisphosphonate precipitate is detected in the NMR spectra. This observation is in agreement with ^31^P solution-state NMR spectra, which show a decrease of the formed bisphosphonate resonances on a similar time scale. In contrast, the ATP^N^ resonances remain constant in intensity in the CP spectra, indicating that the triphosphate of the dinucleotide remains bound to the HBD. The phosphodiester resonance of the dinucleotide overlaps with bound DNA peaks. A gradual decrease in this region over time is attributed to conformational changes in the bound DNA upon primer synthesis. Despite the significant differences of protein concentration in the sediment and in the NMR tube, the kinetics for the initial primer formation are rather similar. Neither in the sedimented primase, nor in solution, is a complete conversion of the educts observed.

Proton-detected solid-state NMR has allowed us to monitor the primer synthesis in a sedimented protein sample by detecting protein conformational changes. In contrast to ^13^C-detected experiments in MAS rotors with a larger diameter (e.g., 3.2 mm rotors requiring ∼25 mg of protein sample), the experiments in the 0.7 mm probe benefit from (i) requiring much less sample (∼0.5 mg) and (ii) short acquisition time allowing to record 2D spectra in reasonable time to monitor biochemical reactions. This allows protein conformational changes to be monitored which occur in the HBD based on changes in signals of residues involved in binding the first two cognate nucleotides required for dinucleotide formation. Resonance changes are detected for amino acids located in the ATP-binding sites previously reported in the ATP-bound state,^13^ namely ATP1 (R309, S310, R311 and H315) and ATP2 (N348 and E345).

## Conclusions

The combination of phosphorus-31 detected solid- and solution-state NMR experiments allowed us to monitor in real time the formation of the initial dinucleotide in the archaeal primase from the plasmid pRN1. NTP-analogues have been used to slow the reaction for such purposes. Primer synthesis also occurs in a sedimented protein sample (∼450 mg/mL protein concentration), and the kinetics are comparable to those determined by solution-state NMR. Our data reveal that the HBD domain of the primase binds both substrates necessary for dinucleotide formation before handing them to the catalytic domain. Protein conformational changes during this reaction were investigated by solid-state NMR experiments at fast MAS. Such solid-state NMR studies are of particular interest for oligomeric proteins and their complexes or large proteins in general, whose NMR spectra in solution are significantly influenced by lifetime broadening effects that in contrast do not affect the spectra in the solid state.

## Experimental Section

### Protein Expression and Purification

The helix-bundle domain (HBD, residues 256–370), the catalytic domain (residues 40–248) and the functional pRN1 primase (residues 40–370) were expressed and purified using the same method as descripted before.^13^ Briefly, *E. coli* BL21 (DE3) Codon+ carrying the primase plasmid were transformed and grown at 37 °C until an OD_600_ of 0.6–0.7 was reached and then induced with 1 mM IPTG at 30 °C. For the production of unlabeled protein, cells were grown in LB medium. To produce uniformly labeled pRN1 primase, cells were grown in M9 minimal medium containing 1 g/L ^15^NH_4_Cl and further supplemented with 4 g/L glucose (or 2 g/L of ^13^C-glucose for double-labeled pRN1 primase). For ^2^H-labeled protein, the M9 minimal medium was prepared in D_2_O. The cells were harvested and lysed by cell cracker in 50 mM Tris, pH 8.0, 1 M NaCl and 0.1%(v/v) Triton X-100. After centrifugation (34 000g, 40 min), the extracts were loaded onto a HiTrap IMAC FF column charged with Ni^2+^. The column was equilibrated with 50 mM Tris, pH 8.0, 1 M NaCl and 10 mM imidazole. The protein was eluted with a linear imidazole gradient at about 120 mM imidazole. The imidazole was removed by dialysis, and the protein was further purified by gel filtration. Peak fractions were pooled and concentrated between 0.5 and 4.6 mM. The proteins were stored in 50 mM MOPS, pH 7.0, 50 mM NaCl.

### Solid-State NMR Sample Formation

For the primase-DNA^CT^-ATP^N^-dGTP^C^ complex formation, we first mixed primase, DNA^CT^ and ATP^N^ in 50 mM MOPS pH 7.0, 50 mM NaCl, and 10 mM MgCl_2_ with a 1:1.1:2 molar ratio for [primase]:[DNA^CT^]:[ATP^N^]. The complex of primase-DNA^CT^-ATP^N^ was incubated on ice for 30 min, and then the dGTP^C^ was added to form the primase-DNA^C^-ATP^N^-dGTP^C^ complex with a molar ratio of 1:1.1:2:2. The primase-DNA^CT^-ATP^C^-dGTP^C^ and primase-H145A- DNA^CT^-ATP^N^-dGTP^C^ complexes were prepared in the same way. All samples for the solid-state NMR were prepared in 50 mM MOPS pH 7.0, 50 mM NaCl and 10 mM MgCl_2_. All protein solutions were sedimented^33,34,67^ in the MAS NMR rotor (16 h at 4 °C at 210,000 × g) using home-built rotor-filling tools.^70^

### ^31^P Solution-State NMR

^31^P solution-state NMR experiments were performed using a 0.5 mM sample of unlabeled primase-DNA^CT^-ATP^N^-dGTP^C^ in NMR buffer (50 mM MOPS, pH 7.0, 50 mM NaCl, 10 mM MgCl_2_ and 10% D_2_O) at 281 K in a 3 mm diameter NMR tube. The molar ratio of [primase]:[DNA^CT^]:[ATP^N^]:[dGTP^C^] was 1:1:2:2. The primase-DNA^CT^-ATP^N^ complex was first prepared and used to set up the NMR parameters. To initiate the reaction, dGTP^C^ was added directly to the sample tube from concentrated stock solutions, and the time-resolved ^31^P NMR spectra were recorded immediately. The spectra were acquired on a 500 MHz Avance NEO spectrometer equipped with a CP-QCI ^1^H/^19^F–^13^C/^15^N/^31^P–^2^H ZGrad probe (Bruker) using the software package Topspin (versions 4.0.7). The spectra consisted of 2048 complex points with a spectral width of 49 ppm. The scan number was 20480, and each 1D ^31^P spectrum took about 6.5 h.

### NTP-Analogues Binding to pRN1 Primase Studied by Solution-State NMR

To examine how the HBD binds NTP and its analogues, each time dGTP, GTP or a GTP analogue was added to the complex of HBD-DNA^CT^-ATP with 0.1 mM ^15^N-labeled HBD at 298 K, with a molar ratio of [HBD]:[DNA^CT^]:[ATP]:[GTP analogues] at 1:1.1:4:4. The NMR buffer used was 25 mM NaH_2_PO4/Na_2_HPO_4_, pH 7.0, 75 mM NaCl, and 10 mM MgCl_2_.

To investigate how the catalytic domain binds NTP and its analogues, NMR titrations were performed by adding dATP, dATP^C^ or dATP^N^ to ^15^N-labeled protein at 298 K and monitored using ^1^H–^15^N HSQC. The NMR buffer utilized was 25 mM NaH_2_PO4/Na_2_HPO_4_, pH 7.0, 50 mM NaCl, 10 mM MgCl_2_.

### Solid-State NMR

^1^H- and ^31^P-detected solid-state NMR spectra were acquired at 11.7 and 20.0 T static magnetic-field strength using 0.7, 1.3, and 3.2 mm Bruker Biospin probes. The MAS frequency was set to 17, 20, and 100 kHz, respectively. The sample temperature was set between 278 and 309 K using the water line as an internal reference.^70^ The spectra were processed with the software TOPSPIN (version 3.5, Bruker Biospin) with a shifted (2.5 to 3.0) squared cosine apodization function and automated baseline correction in the indirect and direct dimension. An overview of the experimental parameters for all NMR spectra is given in Table S2. Spectra were analyzed with the software CcpNmr^71−73^ (version 2.4.2) and referenced to 3-(trimethylsilyl)propane-1-sulfonate (DSS).

## Supporting Information Available

Supporting figures of additional NMR experiments and experimental details of the presented NMR spectra. (PDF)

## References

[ref1] Frick D. N.; Richardson C. C. DNA primases. Annu. Rev. Biochem. 2001, 70, 39 10.1146/annurev.biochem.70.1.39. 11395402

[ref2] Sugimoto K.; Okazaki T.; Okazaki R. Mechanism of DNA chain growth, II. Accumulation of newly synthesized short chains in E. coli infected with ligase-defective T4 phages. Proc. Natl. Acad. Sci. U. S. A. 1968, 60, 1356 10.1073/pnas.60.4.1356. 4299945 PMC224926

[ref3] Pandey M.; Syed S.; Donmez I.; Patel G.; Ha T.; Patel S. S. Coordinating DNA replication by means of priming loop and differential synthesis rate. Nature 2009, 462, 940 10.1038/nature08611. 19924126 PMC2896039

[ref4] Engler M. J.; Richardson C. C. Bacteriophage T7 DNA replication. Synthesis of lagging strands in a reconstituted system using purified proteins. J. Biol. Chem. 1983, 258, 11197 10.1016/S0021-9258(17)44403-6. 6885817

[ref5] Naue N.; Beerbaum M.; Bogutzki A.; Schmieder P.; Curth U. The helicase-binding domain of Escherichia coli DnaG primase interacts with the highly conserved C-terminal region of single-stranded DNA-binding protein. Nucleic Acids Res. 2013, 41, 4507 10.1093/nar/gkt107. 23430154 PMC3632105

[ref6] Guilliam T. A.; Keen B. A.; Brissett N. C.; Doherty A. J. Primase-polymerases are a functionally diverse superfamily of replication and repair enzymes. Nucleic Acids Res. 2015, 43, 6651 10.1093/nar/gkv625. 26109351 PMC4538821

[ref7] Barry E. R.; Bell S. D. DNA replication in the archaea. Microbiol. Mol. Biol. Rev. 2006, 70, 876 10.1128/MMBR.00029-06. 17158702 PMC1698513

[ref8] Holzer S.; Yan J.; Kilkenny M. L.; Bell S. D.; Pellegrini L. Primer synthesis by a eukaryotic-like archaeal primase is independent of its Fe-S cluster. Nat. Commun. 2017, 8, 1718 10.1038/s41467-017-01707-w. 29167441 PMC5700102

[ref9] Greci M. D.; Dooher J. D.; Bell S. D. The combined DNA and RNA synthetic capabilities of archaeal DNA primase facilitate primer hand-off to the replicative DNA polymerase. Nat. Commun. 2022, 13, 433 10.1038/s41467-022-28093-2. 35064114 PMC8782868

[ref10] Schneider A.; Bergsch J.; Lipps G. The monomeric archaeal primase from Nanoarchaeum equitans harbours the features of heterodimeric archaeoeukaryotic primases and primes sequence-specifically. Nucleic Acids Res. 2023, 51, 5087 10.1093/nar/gkad261. 37099378 PMC10250227

[ref11] Vaithiyalingam S.; Warren E. M.; Eichman B. F.; Chazin W. J. Insights into eukaryotic DNA priming from the structure and functional interactions of the 4Fe-4S cluster domain of human DNA primase. Proc. Natl. Acad. Sci. U. S. A. 2010, 107, 13684 10.1073/pnas.1002009107. 20643958 PMC2922289

[ref12] Baranovskiy A. G.; Zhang Y.; Suwa Y.; Babayeva N. D.; Gu J.; Pavlov Y. I.; Tahirov T. H. Crystal structure of the human primase. J. Biol. Chem. 2015, 290, 5635 10.1074/jbc.M114.624742. 25550159 PMC4342476

[ref13] Boudet J.; Devillier J.-C.; Wiegand T.; Salmon L.; Meier B. H.; Lipps G.; Allain F. H.-T. A Small Helical Bundle Prepares Primer Synthesis by Binding Two Nucleotides that Enhance Sequence-Specific Recognition of the DNA Template. Cell 2019, 176, 154 10.1016/j.cell.2018.11.031. 30595448

[ref14] Baranovskiy A. G.; Babayeva N. D.; Zhang Y. B.; Gu J. Y.; Suwa Y.; Pavlov Y. I.; Tahirov T. H. Mechanism of Concerted RNA-DNA Primer Synthesis by the Human Primosome. J. Biol. Chem. 2016, 291, 10006 10.1074/jbc.M116.717405. 26975377 PMC4858955

[ref15] Keck J. L.; Roche D. D.; Lynch A. S.; Berger J. M. Structure of the RNA polymerase domain of E. coli primase. Science 2000, 287, 2482 10.1126/science.287.5462.2482. 10741967

[ref16] Beck K.; Lipps G. Properties of an unusual DNA primase from an archaeal plasmid. Nucleic Acids Res. 2007, 35, 5635 10.1093/nar/gkm625. 17709343 PMC2034472

[ref17] Kuchta R. D.; Stengel G. Mechanism and evolution of DNA primases. Biochim. Biophys. Acta 2010, 1804, 1180 10.1016/j.bbapap.2009.06.011. 19540940 PMC2846230

[ref18] Li A. W. H.; Zabrady K.; Bainbridge L. J.; Zabrady M.; Naseem-Khan S.; Berger M. B.; Kolesar P.; Cisneros G. A.; Doherty A. J. Molecular basis for the initiation of DNA primer synthesis. Nature 2022, 605, 767 10.1038/s41586-022-04695-0. 35508653 PMC9149119

[ref19] Schneider A.; Smith R. W.; Kautz A. R.; Weisshart K.; Grosse F.; Nasheuer H. P. Primase activity of human DNA polymerase alpha-primase. Divalent cations stabilize the enzyme activity of the p48 subunit. J. Biol. Chem. 1998, 273, 21608 10.1074/jbc.273.34.21608. 9705292

[ref20] Pintér G.; Hohmann K. F.; Grün J. T.; Wirmer-Bartoschek J.; Glaubitz C.; Fürtig B.; Schwalbe H. Real-time nuclear magnetic resonance spectroscopy in the study of biomolecular kinetics and dynamics. Magn. Reson. 2021, 2, 291 10.5194/mr-2-291-2021. PMC10539803 37904763

[ref21] Blanc F.; Leskes M.; Grey C. P. In Situ Solid-State NMR Spectroscopy of Electrochemical Cells: Batteries, Supercapacitors, and Fuel Cells. Acc. Chem. Res. 2013, 46, 1952 10.1021/ar400022u. 24041242

[ref22] Xu M.; Harris K. D. M. Altering the Polymorphic Product Distribution in a Solid-State Dehydration Process by Rapid Sample Rotation in a Solid-State NMR Probe. J. Am. Chem. Soc. 2005, 127, 10832 10.1021/ja052668p. 16076180

[ref23] Xu Y.; Champion L.; Gabidullin B.; Bryce D. L. A kinetic study of mechanochemical halogen bond formation by in situ ^31^P solid-state NMR spectroscopy. Chem. Commun. 2017, 53, 9930 10.1039/C7CC05051H. 28829065

[ref24] Kaabel S.; Stein R. S.; Fomitšenko M.; Järving I.; Friščić T.; Aav R. Size-Control by Anion Templating in Mechanochemical Synthesis of Hemicucurbiturils in the Solid State. Angew. Chem., Int. Ed. 2019, 58, 6230 10.1002/anie.201813431. 30664335

[ref25] Mandala V. S.; Loewus S. J.; Mehta M. A. Monitoring Cocrystal Formation via In Situ Solid-State NMR. J. Phys. Chem. Lett. 2014, 5, 3340 10.1021/jz501699h. 26278442

[ref26] Silva I. d. A. A.; Bartalucci E.; Bolm C.; Wiegand T. Opportunities and Challenges in Applying Solid-State NMR Spectroscopy in Organic Mechanochemistry. Adv. Mater. 2023, 35, 2304092 10.1002/adma.202304092. 37407000

[ref27] Bertini I.; Gallo G.; Korsak M.; Luchinat C.; Mao J.; Ravera E. Formation Kinetics and Structural Features of Beta-Amyloid Aggregates by Sedimented Solute NMR. ChemBioChem. 2013, 14, 1891 10.1002/cbic.201300141. 23821412

[ref28] Jeon J.; Yau W.-M.; Tycko R. Early events in amyloid-β self-assembly probed by time-resolved solid state NMR and light scattering. Nat. Commun. 2023, 14, 2964 10.1038/s41467-023-38494-6. 37221174 PMC10205749

[ref29] Falkenstein P.; Zhao Z.; Di Pede-Mattatelli A.; Künze G.; Sommer M.; Sonnendecker C.; Zimmermann W.; Colizzi F.; Matysik J.; Song C. On the Binding Mode and Molecular Mechanism of Enzymatic Polyethylene Terephthalate Degradation. ACS Catal. 2023, 13, 6919 10.1021/acscatal.3c00259.

[ref30] Tekwani Movellan K.; Wegstroth M.; Overkamp K.; Leonov A.; Becker S.; Andreas L. B. Real-time tracking of drug binding to influenza A M2 reveals a high energy barrier. J. Struct. Biol. X 2023, 8, 100090 10.1016/j.yjsbx.2023.100090. 37363040 PMC10285276

[ref31] Nami F.; Ferraz M. J.; Bakkum T.; Aerts J. M. F. G.; Pandit A. Real-Time NMR Recording of Fermentation and Lipid Metabolism Processes in Live Microalgae Cells. Angew. Chem., Int. Ed. 2022, 61, e202117521 10.1002/anie.202117521. PMC9305762 35103372

[ref32] Suzuki Y.; Morie S.; Okamura H.; Asakura T.; Naito A. Real-Time Monitoring of the Structural Transition of Bombyx mori Liquid Silk under Pressure by Solid-State NMR. J. Am. Chem. Soc. 2023, 145, 22925 10.1021/jacs.3c04361. 37828719

[ref33] Bertini I.; Luchinat C.; Parigi G.; Ravera E.; Reif B.; Turano P. Solid-state NMR of proteins sedimented by ultracentrifugation. Proc. Natl. Acad. Sci. U. S. A. 2011, 108, 10396 10.1073/pnas.1103854108. 21670262 PMC3127869

[ref34] Gardiennet C.; Schütz A. K.; Hunkeler A.; Kunert B.; Terradot L.; Böckmann A.; Meier B. H. A Sedimented Sample of a 59 kDa Dodecameric Helicase Yields High-Resolution Solid-State NMR Spectra. Angew. Chem., Int. Ed. 2012, 51, 7855 10.1002/anie.201200779. 22740125

[ref35] Wiegand T.; Lacabanne D.; Torosyan A.; Boudet J.; Cadalbert R.; Allain F. H. T.; Meier B. H.; Böckmann A. Sedimentation Yields Long-Term Stable Protein Samples as Shown by Solid-State NMR. Front. Mol. Biosci. 2020, 7, 17 10.3389/fmolb.2020.00017. 32154263 PMC7047159

[ref36] le Paige U. B.; Xiang S.; Hendrix M. M. R. M.; Zhang Y.; Folkers G. E.; Weingarth M.; Bonvin A. M. J. J.; Kutateladze T. G.; Voets I. K.; Baldus M.; van Ingen H. Characterization of nucleosome sediments for protein interaction studies by solid-state NMR spectroscopy. Magn. Reson. 2021, 2, 187 10.5194/mr-2-187-2021. PMC9135053 35647606

[ref37] Hellmich U. A.; Haase W.; Velamakanni S.; van Veen H. W.; Glaubitz C. Caught in the act: ATP hydrolysis of an ABC-multidrug transporter followed by real-time magic angle spinning NMR. FEBS Lett. 2008, 582, 3557 10.1016/j.febslet.2008.09.033. 18817774

[ref38] Rydzek S.; Shein M.; Bielytskyi P.; Schütz A. K. Observation of a Transient Reaction Intermediate Illuminates the Mechanochemical Cycle of the AAA-ATPase p97. J. Am. Chem. Soc. 2020, 142, 14472 10.1021/jacs.0c03180. 32790300

[ref39] Kaur H.; Lakatos-Karoly A.; Vogel R.; Nöll A.; Tampé R.; Glaubitz C. Coupled ATPase-adenylate kinase activity in ABC transporters. Nat. Commun. 2016, 7, 13864 10.1038/ncomms13864. 28004795 PMC5192220

[ref40] Ullrich S. J.; Hellmich U. A.; Ullrich S.; Glaubitz C. Interfacial enzyme kinetics of a membrane bound kinase analyzed by real-time MAS-NMR. Nat. Chem. Biol. 2011, 7, 263 10.1038/nchembio.543. 21423170

[ref41] de Mos J.; Jakob A.; Becker-Baldus J.; Heckel A.; Glaubitz C. Light-Induced Uncaging for Time-Resolved Observations of Biochemical Reactions by MAS NMR Spectroscopy. Chem.—Eur. J. 2020, 26, 6789 10.1002/chem.202000770. 32240561 PMC7317521

[ref42] Lipps G.; Röther S.; Hart C.; Krauss G. A novel type of replicative enzyme harbouring ATPase, primase and DNA polymerase activity. EMBO J. 2003, 22, 2516 10.1093/emboj/cdg246. 12743045 PMC156004

[ref43] Beck K.; Vannini A.; Cramer P.; Lipps G. The archaeo-eukaryotic primase of plasmid pRN1 requires a helix bundle domain for faithful primer synthesis. Nucleic Acids Res. 2010, 38, 6707 10.1093/nar/gkq447. 20511586 PMC2965215

[ref44] Lipps G. Structure and function of the primase domain of the replication protein from the archaeal plasmid pRN1. Biochem. Soc. Trans. 2011, 39, 104 10.1042/BST0390104. 21265755

[ref45] Bagshaw C. R. ATP analogues at a glance. J. Cell Sci. 2001, 114, 459 10.1242/jcs.114.3.459. 11171313

[ref46] Lacabanne D.; Wiegand T.; Wili N.; Kozlova M. I.; Cadalbert R.; Klose D.; Mulkidjanian A. Y.; Meier B. H.; Böckmann A. ATP Analogues for Structural Investigations: Case Studies of a DnaB Helicase and an ABC Transporter. Molecules 2020, 25, 5268 10.3390/molecules25225268. 33198135 PMC7698047

[ref47] Wiegand T. A solid-state NMR tool box for the investigation of ATP-fueled protein engines. Prog. Nucl. Magn. Reson. Spectrosc. 2020, 117, 1 10.1016/j.pnmrs.2020.02.001. 32471533

[ref48] Brown G. C. Total cell protein concentration as an evolutionary constraint on the metabolic control distribution in cells. J. Theor. Biol. 1991, 153, 195 10.1016/S0022-5193(05)80422-9. 1787736

[ref49] Stöppler D.; Macpherson A.; Smith-Penzel S.; Basse N.; Lecomte F.; Deboves H.; Taylor R. D.; Norman T.; Porter J.; Waters L. C.; Westwood M.; Cossins B.; Cain K.; White J.; Griffin R.; Prosser C.; Kelm S.; Sullivan A. H.; Fox D. III; Carr M. D.; Henry A.; Taylor R.; Meier B. H.; Oschkinat H.; Lawson A. D. Insight into small molecule binding to the neonatal Fc receptor by X-ray crystallography and 100 kHz magic-angle-spinning NMR. PLOS Biology 2018, 16, e2006192 10.1371/journal.pbio.2006192. 29782488 PMC5983862

[ref50] Agarwal V.; Penzel S.; Szekely K.; Cadalbert R.; Testori E.; Oss A.; Past J.; Samoson A.; Ernst M.; Böckmann A.; Meier B. H. De Novo 3D Structure Determination from Sub-milligram Protein Samples by Solid-State 100 kHz MAS NMR Spectroscopy. Angew. Chem., Int. Ed. 2014, 53, 12253 10.1002/anie.201405730. 25225004

[ref51] Andreas L. B.; Jaudzems K.; Stanek J.; Lalli D.; Bertarello A.; Le Marchand T.; Cala-De Paepe D.; Kotelovica S.; Akopjana I.; Knott B.; Wegner S.; Engelke F.; Lesage A.; Emsley L.; Tars K.; Herrmann T.; Pintacuda G. Structure of fully protonated proteins by proton-detected magic-angle spinning NMR. Proc. Natl. Acad. Sci. U. S. A. 2016, 113, 9187 10.1073/pnas.1602248113. 27489348 PMC4995937

[ref52] Grohe K.; Nimerovsky E.; Singh H.; Vasa S. K.; Söldner B.; Vögeli B.; Rienstra C. M.; Linser R. Exact distance measurements for structure and dynamics in solid proteins by fast-magic-angle-spinning NMR. Chem. Commun. 2019, 55, 7899 10.1039/C9CC02317H. PMC6658100 31199417

[ref53] Linser R.; Bardiaux B.; Andreas L. B.; Hyberts S. G.; Morris V. K.; Pintacuda G.; Sunde M.; Kwan A. H.; Wagner G. Solid-State NMR Structure Determination from Diagonal-Compensated, Sparsely Nonuniform-Sampled 4D Proton-Proton Restraints. J. Am. Chem. Soc. 2014, 136, 11002 10.1021/ja504603g. 24988008 PMC4132958

[ref54] Friedrich D.; Perodeau J.; Nieuwkoop A. J.; Oschkinat H. MAS NMR detection of hydrogen bonds for protein secondary structure characterization. J. Biomol. NMR 2020, 74, 247 10.1007/s10858-020-00307-z. 32185644 PMC7211791

[ref55] Retel J. S.; Nieuwkoop A. J.; Hiller M.; Higman V. A.; Barbet-Massin E.; Stanek J.; Andreas L. B.; Franks W. T.; van Rossum B.-J.; Vinothkumar K. R.; Handel L.; de Palma G. G.; Bardiaux B.; Pintacuda G.; Emsley L.; Kühlbrandt W.; Oschkinat H. Structure of outer membrane protein G in lipid bilayers. Nat. Commun. 2017, 8, 2073 10.1038/s41467-017-02228-2. 29233991 PMC5727033

[ref56] Struppe J.; Quinn C. M.; Lu M.; Wang M.; Hou G.; Lu X.; Kraus J.; Andreas L. B.; Stanek J.; Lalli D.; Lesage A.; Pintacuda G.; Maas W.; Gronenborn A. M.; Polenova T. Expanding the horizons for structural analysis of fully protonated protein assemblies by NMR spectroscopy at MAS frequencies above 100 kHz. Solid State Nucl. Magn. Reson. 2017, 87, 117 10.1016/j.ssnmr.2017.07.001. 28732673 PMC5824719

[ref57] Lalli D.; Idso M. N.; Andreas L. B.; Hussain S.; Baxter N.; Han S.; Chmelka B. F.; Pintacuda G. Proton-Based Structural Analysis of a Heptahelical Transmembrane Protein in Lipid Bilayers. J. Am. Chem. Soc. 2017, 139, 13006 10.1021/jacs.7b05269. 28724288 PMC5741281

[ref58] Jain M. G.; Lalli D.; Stanek J.; Gowda C.; Prakash S.; Schwarzer T. S.; Schubeis T.; Castiglione K.; Andreas L. B.; Madhu P. K.; Pintacuda G.; Agarwal V. Selective 1H-1H Distance Restraints in Fully Protonated Proteins by Very Fast Magic-Angle Spinning Solid-State NMR. J. Phys. Chem. Lett. 2017, 8, 2399 10.1021/acs.jpclett.7b00983. 28492324

[ref59] Le Marchand T.; Schubeis T.; Bonaccorsi M.; Paluch P.; Lalli D.; Pell A. J.; Andreas L. B.; Jaudzems K.; Stanek J.; Pintacuda G. ^1^H-Detected Biomolecular NMR under Fast Magic-Angle Spinning. Chem. Rev. 2022, 122, 9943 10.1021/acs.chemrev.1c00918. 35536915 PMC9136936

[ref60] Nishiyama Y.; Hou G.; Agarwal V.; Su Y.; Ramamoorthy A. Ultrafast Magic Angle Spinning Solid-State NMR Spectroscopy: Advances in Methodology and Applications. Chem. Rev. 2023, 123, 918 10.1021/acs.chemrev.2c00197. 36542732 PMC10319395

[ref61] Lipps G.; Weinzierl A. O.; von Scheven G.; Buchen C.; Cramer P. Structure of a bifunctional DNA primase-polymerase. Nat. Struct. Mol. Biol. 2004, 11, 157 10.1038/nsmb723. 14730355

[ref62] Takegoshi K.; Nakamura S.; Terao T. 13C-1H dipolar-assisted rotational resonance in magic-angle spinning NMR. Chem. Phys. Lett. 2001, 344, 631 10.1016/S0009-2614(01)00791-6.

[ref63] Takegoshi K.; Nakamura S.; Terao T. 13C-13C polarization transfer by resonant interference recoupling under magic-angle spinning in solid-state NMR. Chem. Phys. Lett. 1999, 307, 295 10.1016/S0009-2614(99)00533-3.

[ref64] Mukherjee S.; Song Y.; Oldfield E. NMR Investigations of the Static and Dynamic Structures of Bisphosphonates on Human Bone: a Molecular Model. J. Am. Chem. Soc. 2008, 130, 1264 10.1021/ja0759949. 18173269

[ref65] Mori Y.; Nagamine K.; Tomita N.; Notomi T. Detection of Loop-Mediated Isothermal Amplification Reaction by Turbidity Derived from Magnesium Pyrophosphate Formation. Biochem. Biophys. Res. Commun. 2001, 289, 150 10.1006/bbrc.2001.5921. 11708792

[ref66] Shopsowitz K. E.; Roh Y. H.; Deng Z. J.; Morton S. W.; Hammond P. T. RNAi-Microsponges Form through Self-Assembly of the Organic and Inorganic Products of Transcription. Small 2014, 10, 1623 10.1002/smll.201302676. 24851252 PMC4031615

[ref67] Wiegand T.; Lacabanne D.; Torosyan A.; Boudet J.; Cadalbert R.; Allain F. H.; Meier B. H.; Bockmann A. Sedimentation Yields Long-Term Stable Protein Samples as Shown by Solid-State NMR. Front. Mol. Biosci. 2020, 7, 17 10.3389/fmolb.2020.00017. 32154263 PMC7047159

[ref68] Kirk B. W.; Kuchta R. D. Human DNA primase: Anion inhibition, manganese stimulation, and their effects on in vitro start-site selection. Biochemistry 1999, 38, 10126 10.1021/bi990351u. 10433721

[ref69] Lacabanne D.; Fogeron M.-L.; Wiegand T.; Cadalbert R.; Meier B. H.; Böckmann A. Protein sample preparation for solid-state NMR investigations. Prog. Nucl. Magn. Reson. Spectrosc. 2019, 110, 20 10.1016/j.pnmrs.2019.01.001. 30803692

[ref70] Böckmann A.; Gardiennet C.; Verel R.; Hunkeler A.; Loquet A.; Pintacuda G.; Emsley L.; Meier B.; Lesage A. Characterization of different water pools in solid-state NMR protein samples. J. Biomol. NMR 2009, 45, 319 10.1007/s10858-009-9374-3. 19779834

[ref71] Fogh R.; Ionides J.; Ulrich E.; Boucher W.; Vranken W.; Linge J. P.; Habeck M.; Rieping W.; Bhat T. N.; Westbrook J.; Henrick K.; Gilliland G.; Berman H.; Thornton J.; Nilges M.; Markley J.; Laue E. The CCPN project: an interim report on a data model for the NMR community. Nat. Struct. Mol. Biol. 2002, 9, 416 10.1038/nsb0602-416. 12032555

[ref72] Vranken W. F.; Boucher W.; Stevens T. J.; Fogh R. H.; Pajon A.; Llinas M.; Ulrich E. L.; Markley J. L.; Ionides J.; Laue E. D. The CCPN data model for NMR spectroscopy: Development of a software pipeline. Proteins: Struct., Funct., Bioinf. 2005, 59, 687 10.1002/prot.20449. 15815974

[ref73] Stevens T.; Fogh R.; Boucher W.; Higman V.; Eisenmenger F.; Bardiaux B.; van Rossum B.-J.; Oschkinat H.; Laue E. A software framework for analysing solid-state MAS NMR data. J. Biomol. NMR 2011, 51, 437 10.1007/s10858-011-9569-2. 21953355 PMC3222832

