# Supplementary material for: Initial Primer Synthesis of a DNA Primase Monitored by Real-Time NMR Spectroscopy
Source: J Am Chem Soc. 2024 Mar 27;146(14):9583–96. doi: 10.1021/jacs.3c11836 (PMC11009956; doi:10.1021/jacs.3c11836)
Supplement: Supplementary file 1 — ja3c11836_si_001.pdf [file ja3c11836_si_001.pdf]

Supporting Information for

Initial primer synthesis of a DNA primase monitored by real-time NMR spectroscopy

Pengzhi Wu[a,#], Johannes Zehnder[b,#], Nina Schröder[c,#], Pascal E. W. Blümmel[a], Loïc Salmon[a], Fred. F. Damberger[a], Georg Lipps[d], Frédéric H.-T. Allain[a,*], and Thomas Wiegand[b,c,e,*]

[a] *Department of Biology, Institute of Biochemistry, ETH Zürich, 8093 Zurich, Switzerland*

[b] *Laboratory of Physical Chemistry, ETH Zürich, 8093 Zurich, Switzerland*

[c] *Institute of Technical and Macromolecular Chemistry, RWTH Aachen University, Worringerweg 2, 52074 Aachen, Germany*

[d] *Institute of Chemistry and Bioanalytics, University of Applied Sciences Northwestern Switzerland, Hofackerstrasses 30, 4132 Muttenz, Switzerland*

[e] *Max-Planck-Institute for Chemical Energy Conversion, Stiftstr. 34-36, 45470 Mülheim an der Ruhr, Germany*

#: Equal contributions

*: Corresponding authors: allain@bc.biol.ethz.ch, thomas.wiegand@cec.mpg.de

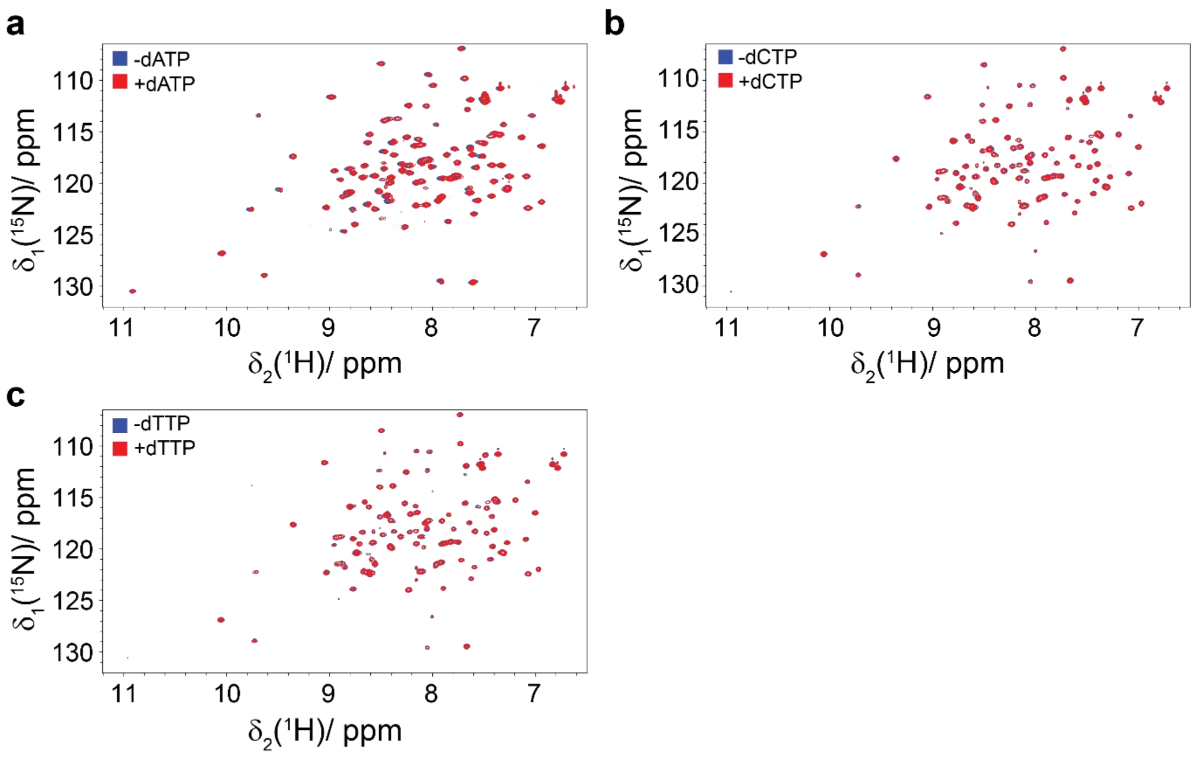

Figure S1: Overlay of ^{1}H-^{15}N solution-state HSQC spectra of HBD-DNA^{CT}-2ATP in the absence (shown in blue) and presence of dATP at pH 7.0 (a), dCTP at pH 5.5 (b), or dTTP at pH 5.5 (c) (shown in red), respectively.

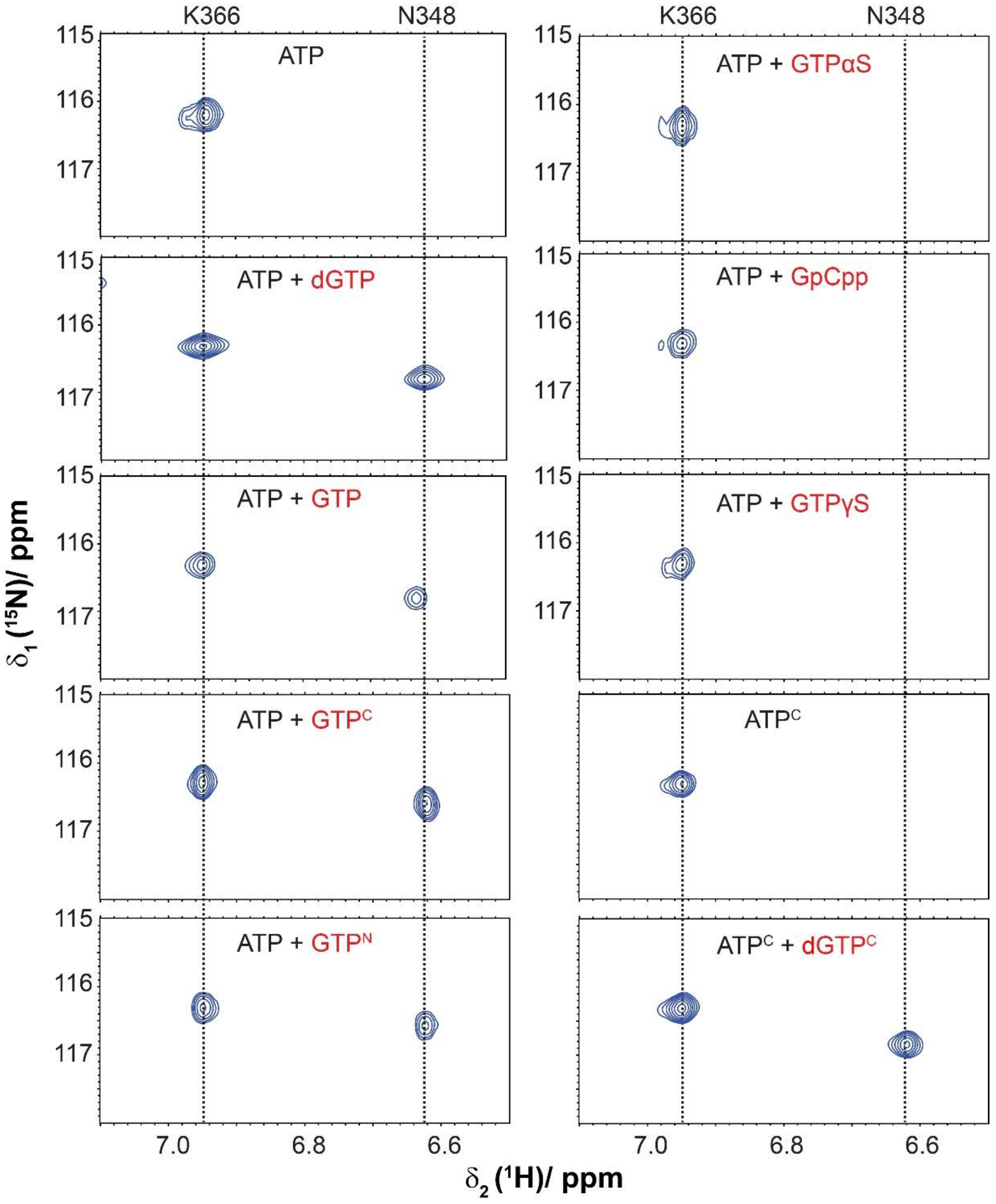

Figure S2: Close-up view of K366 and N348 in a ^{1}H-^{15}N solution-state HSQC experiment with different ATP and dGTP analogues. The cross peak corresponding to N348 becomes only visible, if both nucleotides are bound to the protein, as the loop K340-N348 only rigidifies upon nucleotide binding. This experiment has been used to identify suitable ATP and dGTP analogues for this study.

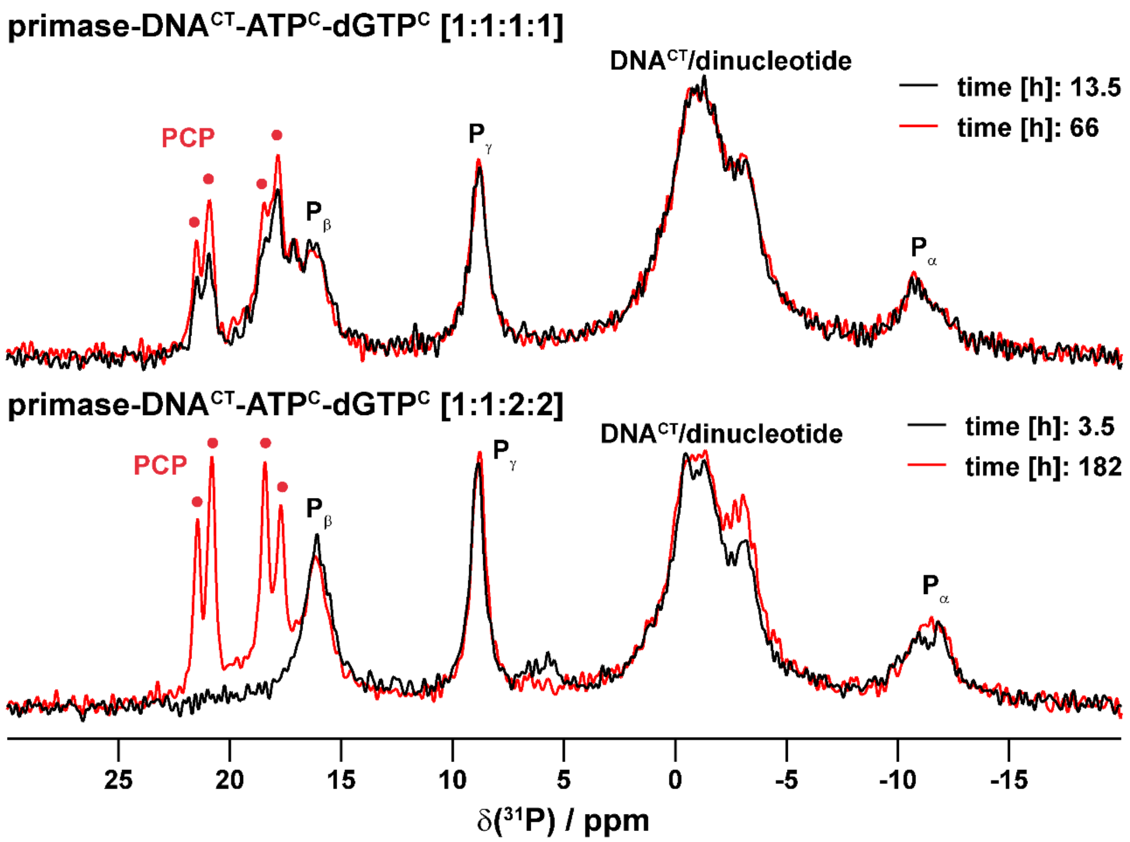

Figure S3: ^{1}H-^{31}P CP-MAS spectra of primase-DNA^{CT}-ATP^{C}-dGTP^{C} with different ATP^{C} and dGTP^{C} ratios. The spectra on top were recorded on a sample using a primase:dGTP^{C}:ATP^{C} 1:1:1 molar ratio and the bottom spectra on a sample with a primase:dGTP^{C}:ATP^{C} 1:2:2 molar ratio. The color of the spectra reflects the time points after ultracentrifugation at which the spectra have been taken. In the 1:1:1 sample the reaction has completed already during ultracentrifugation (only the dinucleotide-bound state is detected), which is not the case in the 1:2:2 sample. In the latter, an increase in dinucleotide binding is observed over time (increasing resonance at around -3 ppm), whereas the constant triphosphate resonances indicate the continuous replacement of bound triphosphates from the excess in the supernatant (pointing to an unfinished reaction). Time point zero represents the end of the ultracentrifugation after spinning up the MAS rotor.

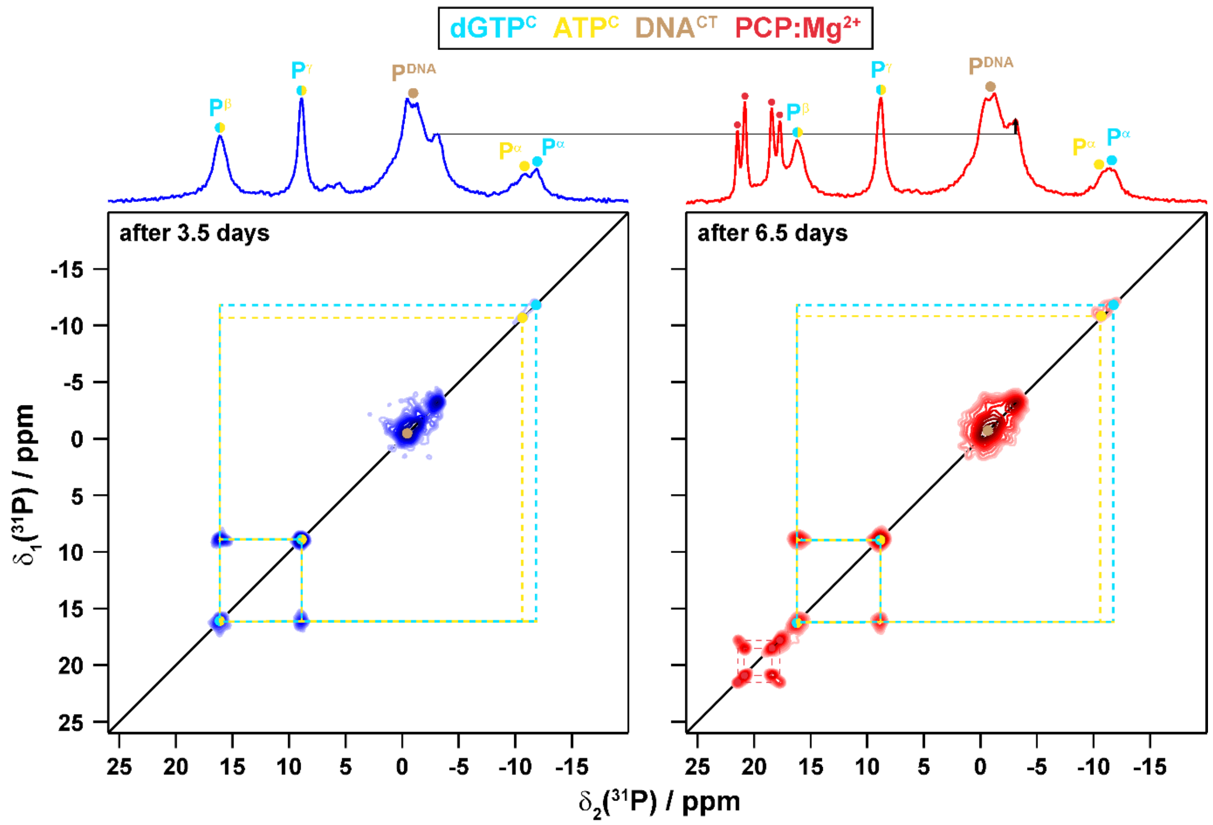

Figure S4: 2D ^{31}P-^{31}P 200 ms PDSD spectra of primase-DNA^{CT}-ATP^{C}-dGTP^{C} before (blue) and after the dinucleotide formation reaction (red). The P^{β} and P^{γ} chemical shifts of ATP^{C} and dGTP^{C} overlap. The formation of the PCP:Mg^{2+} complex and the increase of the peak at around -3 ppm (see black arrow) indicates the formation of the dinucleotide. The P^{α} resonances only appear rather weak on the diagonal with the current signal-to-noise ratio.

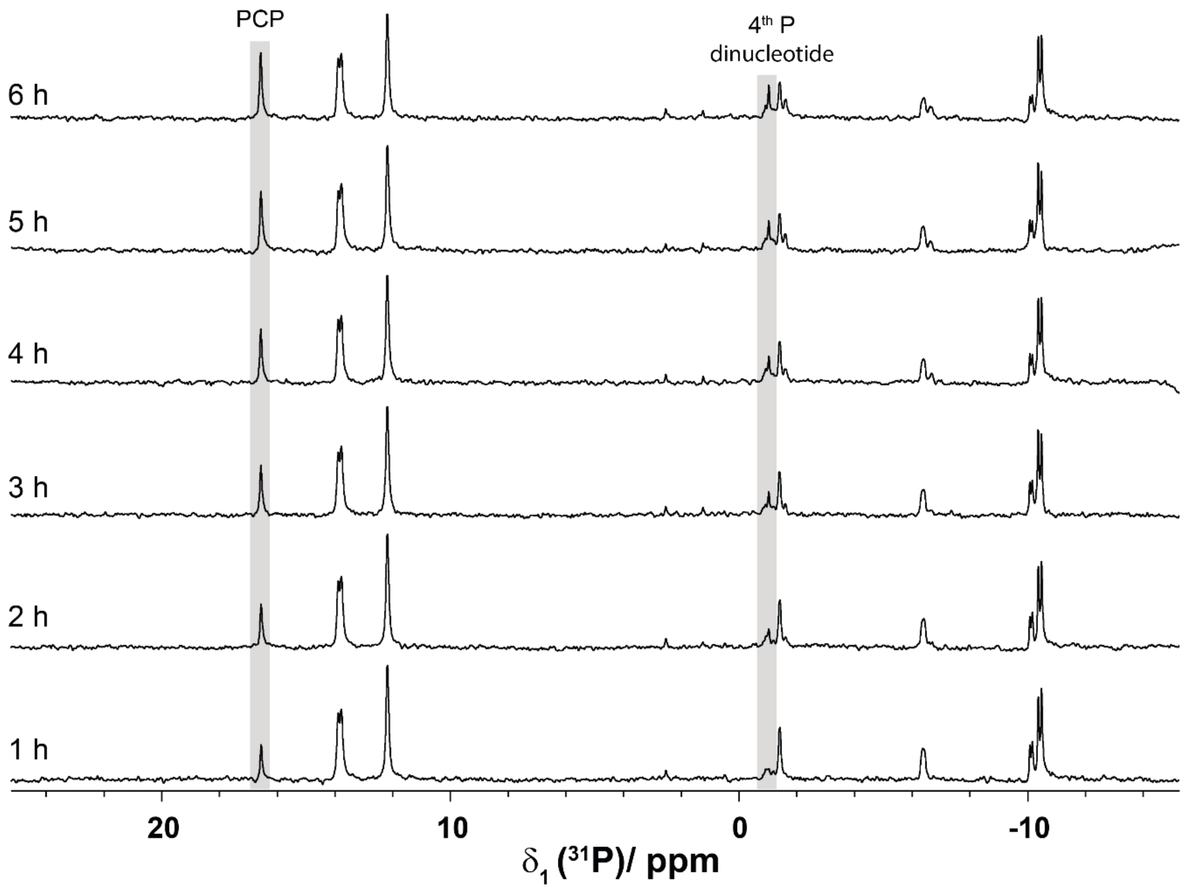

Figure S5: Real-time solution state ^{31}P NMR spectra of primase-DNA^{CT}-ATP^{N}-dGTP^{C} with a 1:1:50:50 ratio. Due to the excess of nucleotides used, the peak corresponding to the 4th phosphate of the dinucleotide (the phosphodiester group) can be distinguished from the DNA template.

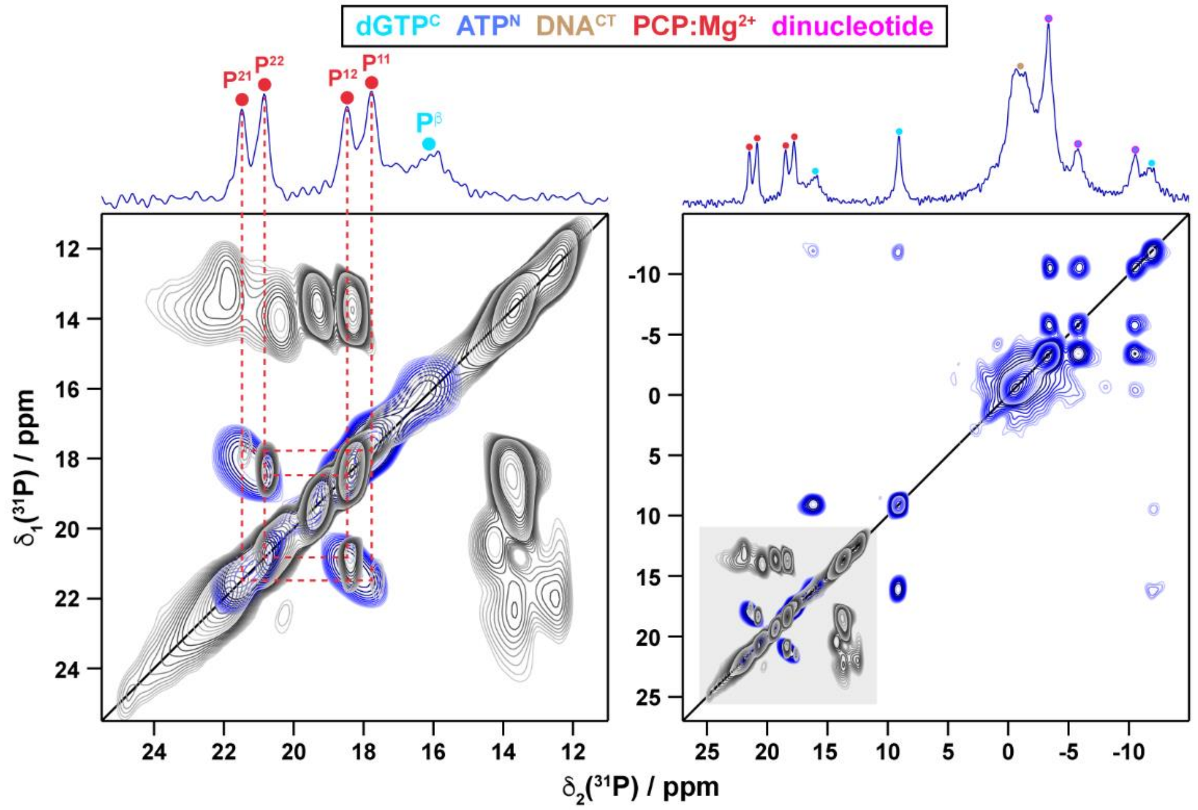

Figure S6: 2D ^{31}P-^{31}P 150 ms DARR spectra of primase-DNA^{CT}-ATP^{N}-dGTP^{C} (blue) and PCP:Mg^{2+} (grey). The latter has been prepared from a solution of medronic acid and $MgCl_2$ (ratio 1:10). A minor fraction of the polymorph of the complex PCP:Mg^{2+} is shown with red dashed lines. Note that the individual PCP resonances are not resolved in the 2D spectrum due to a too short acquisition time in the direct dimension. The additional resonances observed in the grey spectrum are attributed to different polymorphs of PCP:Mg^{2+} formed from the medronic acid solution.

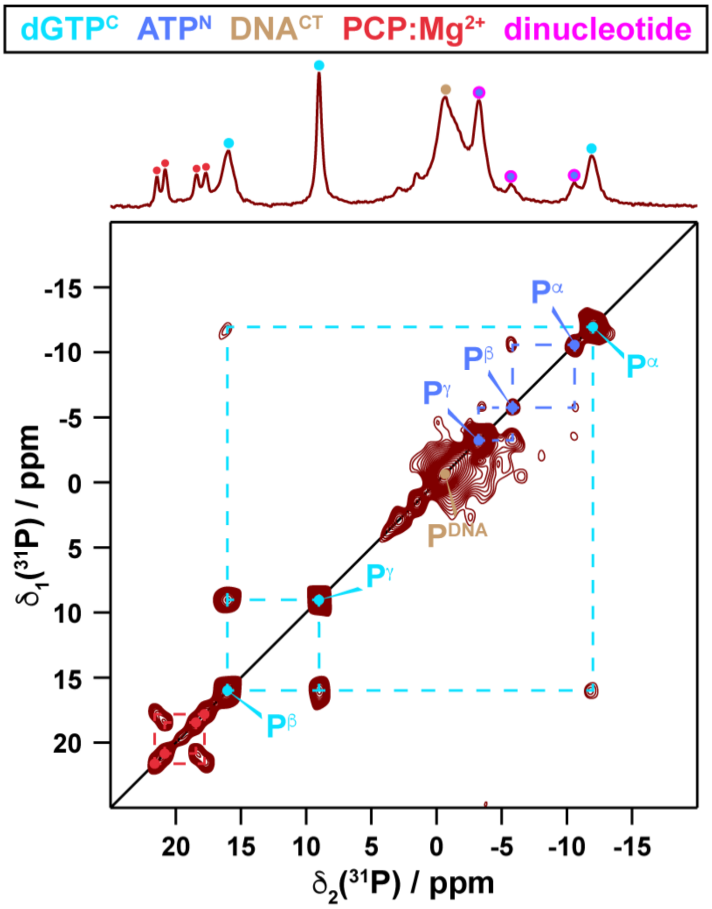

Figure S7: 2D ^{31}P-^{31}P 50 ms DARR spectrum of primase-H145A-DNA^{CT}-ATP^{N}-dGTP^{C}. Dashed lines show the correlation between the neighboring ^{31}P nuclei for ATP^{N}, dGTP^{C} and the PCP:Mg^{2+} complex, in the latter clearly showing the set of two Mg^{2+}:PCP resonances.

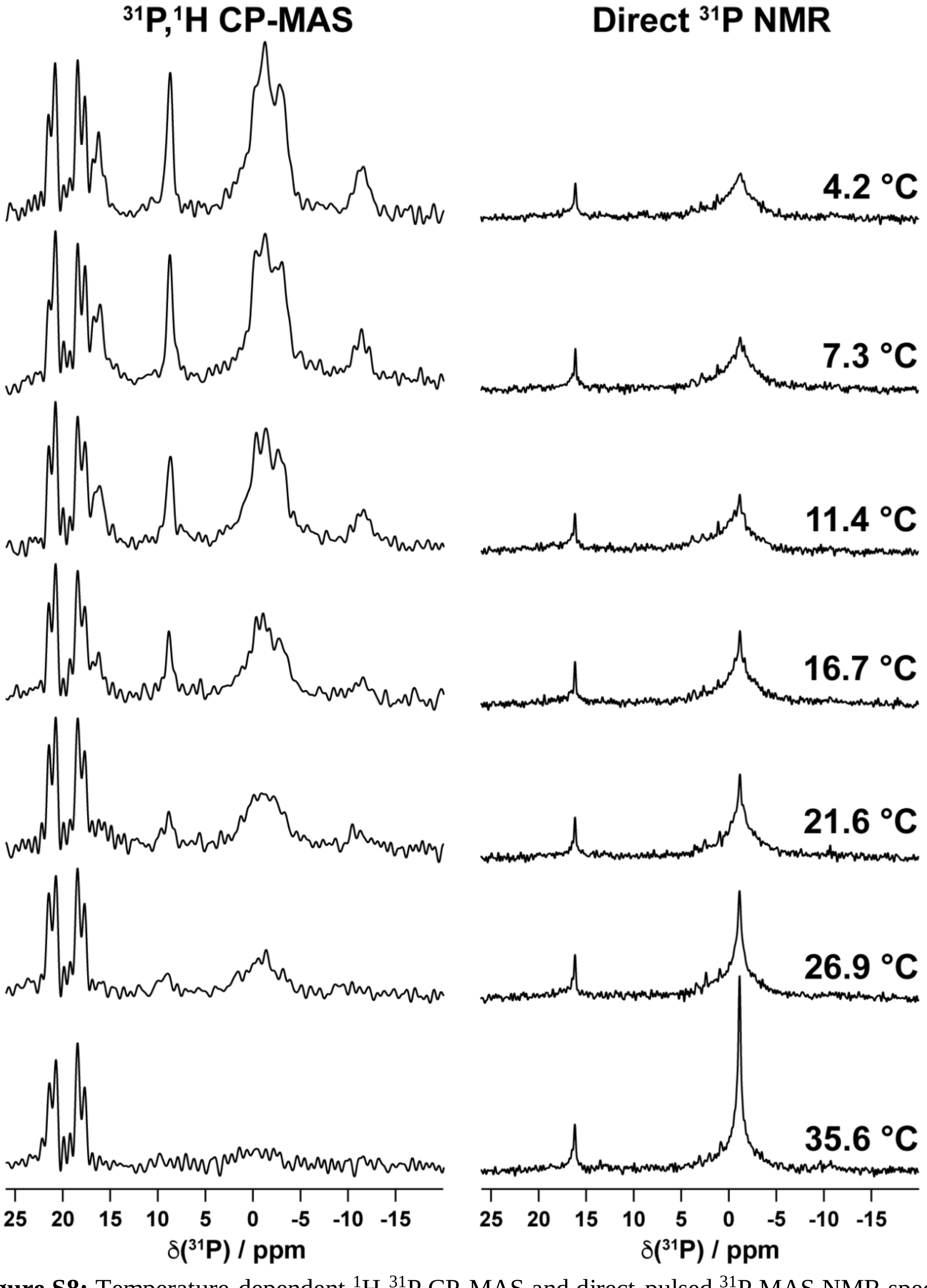

Figure S8: Temperature-dependent ^{1}H-^{31}P CP-MAS and direct-pulsed ^{31}P MAS NMR spectra of primase-DNA^{CT}-ATP^{C}-dGTP^{C}. The bound nucleotides are only detected at low temperature in the CP spectra, whereas at higher temperature only the Mg^{2+}:PCP resonances are observed in the CP spectra.

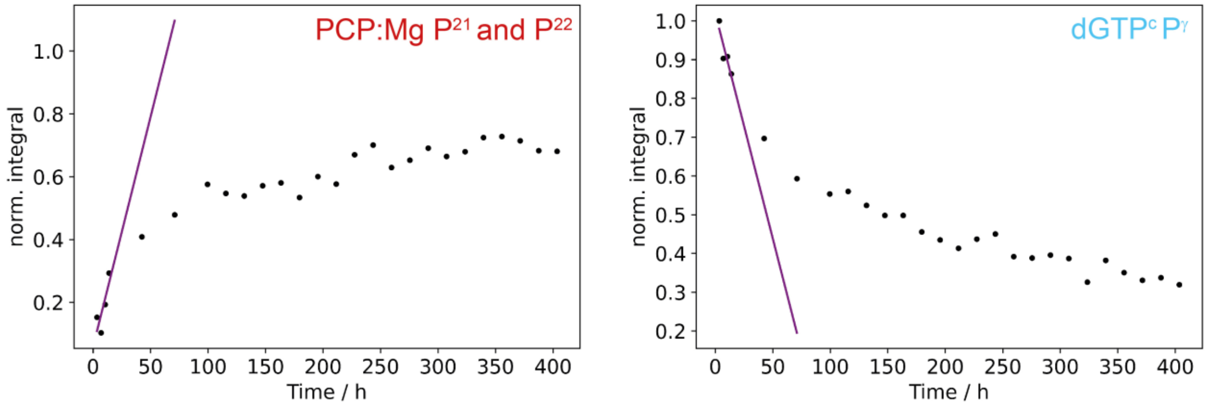

Figure S9: *Changes in the peak integrals of the ^{1}H-^{31}P CP-MAS spectra of primase-DNA^{CT}-ATP^{N}-dGTP^{C} as a function of time.* The Pγ integral of dGTP^{C} has been normalized to 1(right panel), which assumes that the reaction that is occurring before taking the first spectrum is negligible. For PCP the leftmost pair of resonances has been integrated (left panel). The integrals have been normalized with the last data point to 0.68 (which corresponds to the integral of the dGTP^{C} Pγ resonance after ~400 h). This has been done to compensate for the incomplete reaction after more than 400 h. The assumption that the amount of PCP formation before the first measurement is negligible and that the process of normalization is therefore valid, is based on the missing PCP resonances in the first ^{1}H-^{31}P CP-MAS spectrum after rotor filling and has been confirmed by analyzing the ratio of the absolute integrals of dGTP^{C} (t= 0 h)/ PCP (t= 400 h), which is 0.69. With this method, we observe an increase in PCP with an initial rate constant of 0.35 ± 0.17 d^{-1} and a decrease in dGPT^{C} with an initial rate constant of 0.28 ± 0.09 d^{-1}, purple linear regressions in both plots.

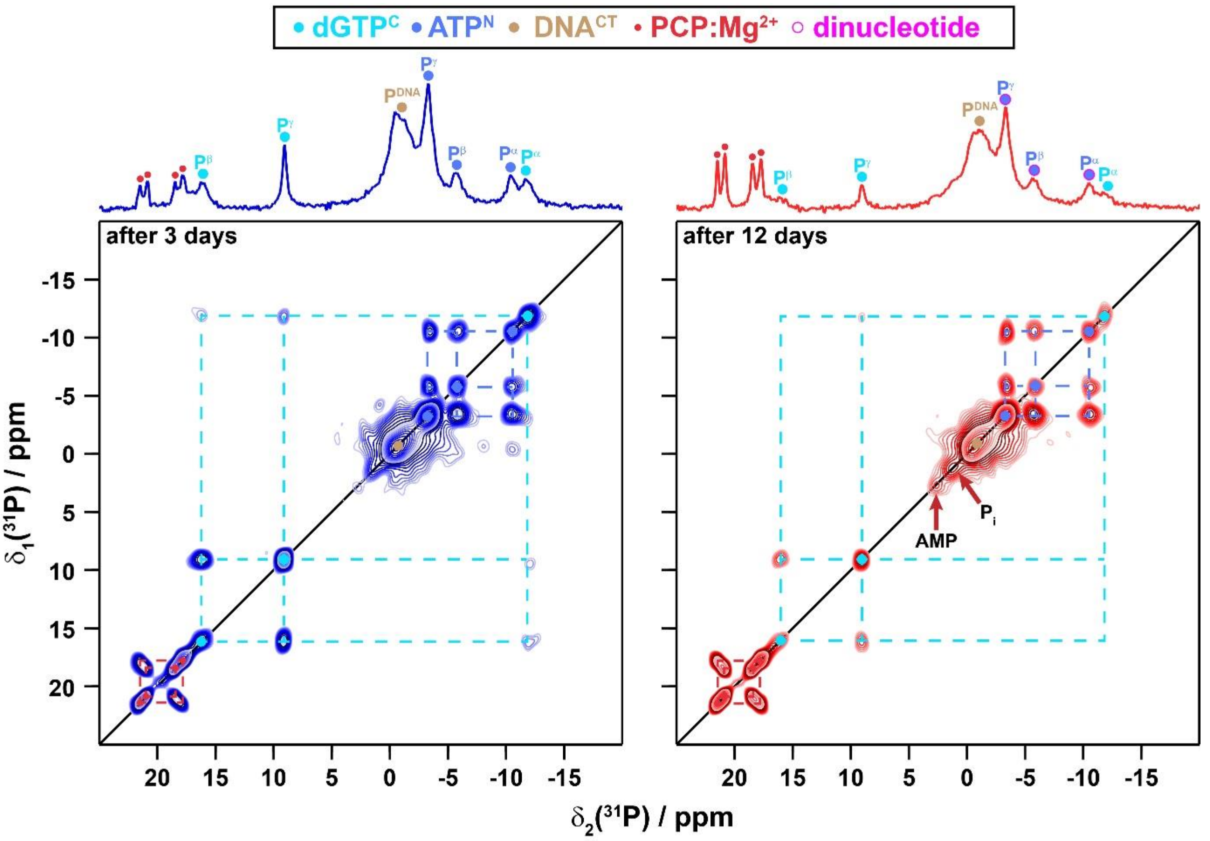

Figure S10: 2D ^{31}P-^{31}P 150 ms DARR spectra of primase-DNA^{CT}-ATP^{N}-dGTP^{C} after 3 days (blue) and 12 days (red) of sample filling. Red arrows show the side products (AMP and P_i) of ATP^{N} hydrolysis.

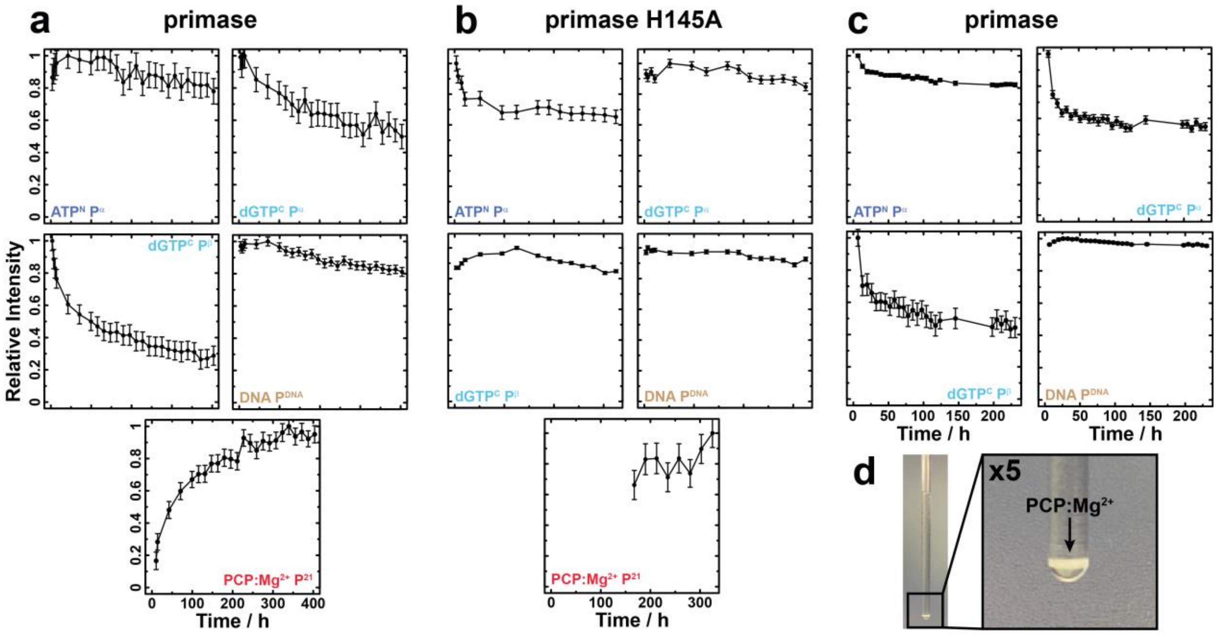

Figure S11: Intensity change as a function of time determined from the ^{1}H-^{31}P CPMAS spectra of primase-DNA^{CT}-ATP^{N}-dGTP^{C} (**a**), from the ^{1}H-^{31}P CPMAS spectra of primase-H145A-DNA^{CT}-ATP^{N}-dGTP^{C} (**b**) and from the solution-state ^{31}P spectra of primase-DNA^{CT}-ATP^{N}-dGTP^{C} (**c**). (**d**) Solution-state NMR tube of primase-DNA^{CT}-ATP^{N}-dGTP^{C} showing the formed Mg^{2+}:PCP complex as white precipitate.

Table S1: CSP data extracted from 2D hNH between primase-H145A-DNA^{CT}-ATP^{N}-dGTP^{C} and primase-DNA^{CT}-ATP^{N}-dGTP^{C} (spectra are shown in Figure 6). CSPs were calculated according to $\sqrt{\Delta\delta_{1H}{}^2 + 0.2 \cdot \Delta\delta_{15N}{}^2}$.

Residue	Cross Peak	CSP / ppm
W49	$(H^{\varepsilon1},N^{\varepsilon1})$	0.06
G54	(H,N)	0.04
N56	(H,N)	0.06
D61	(H,N)	0.12
W72	(H,N)	0.03
W72	$(H^{\varepsilon1},N^{\varepsilon1})$	0.01
G95	(H,N)	0.07
G102	(H,N)	0.03
G103	(H,N)	0.12
Q104	(H,N)	0.07
G106	(H,N)	0.04
L110	(H,N)	0.08
S114	(H,N)	0.03
K115	(H,N)	0.01
W121	$(H^{\varepsilon1},N^{\varepsilon1})$	0.12
L135	(H,N)	0.09
T137	(H,N)	0.18
T139	(H,N)	0.29
G143	(H,N)	0.04
Y147	(H,N)	0.05
V148	(H,N)	0.06
S150	(H,N)	0.07
F162	(H,N)	0.08
E163	(H,N)	0.05
N165	(H,N)	0.05
G166	(H,N)	0.05
G168	(H,N)	0.08
S177	(H,N)	0.22
N187	$(H^{\delta21},N^{\delta2})$	0.07
N187	$(H^{\delta22},N^{\delta2})$	0.12
K199	(H,N)	0.06
N211	(H,N)	0.06
K214	(H,N)	0.25
L221	(H,N)	0.06
G232	(H,N)	0.03
G236	(H,N)	0.05
I237	(H,N)	0.04
A243	(H,N)	0.02
W246	$(H^{\varepsilon1},N^{\varepsilon1})$	0.03
D271	(H,N)	0.10
G273	(H,N)	0.04
E277	(H,N)	0.03
K278	(H,N)	0.07
K286	(H,N)	0.05

N299	(H,N)	0.04
N299	($H^{\delta2b}$,$N^{\delta2}$)	0.05
T301	(H,N)	0.04
Y302	(H,N)	0.04
R309	(H,N)	0.13
R309	(H^{ε},N^{ε})	0.56
S310	(H,N)	0.14
R311	(H,N)	0.19
G312	(H,N)	0.08
W314	($H^{\varepsilon1}$,$N^{\varepsilon1}$)	0.07
H315	($H^{\varepsilon2}$,$N^{\varepsilon2}$)	0.27
G324	(H,N)	0.01
T326	(H,N)	0.05
E333	(H,N)	0.21
L335	(H,N)	0.04
D338	(H,N)	0.23
K340	(H,N)	0.03
N344	(H,N)	0.02
E345	(H,N)	0.27
Q350	(H,N)	0.02
W361	($H^{\varepsilon1}$,$N^{\varepsilon1}$)	0.04
K366	(H,N)	0.08
A370	(H,N)	0.07

Table S2: Overview about experimental parameters of the performed solid-state NMR experiments. For more details about the used adiabatic CP steps and the tangential shapes used see reference [2].

Sample	primase-DNA^{CT}-ATP^{N}-dGTP^{C}	primase-H145A-DNA^{CT}-ATP^{N}-dGTP^{C}	primase-DNA^{CT}-ATP^{N}-dGTP^{C}	primase-H145A-DNA^{CT}-ATP^{N}-dGTP^{C}
Experiment	^{1}H-^{31}P CP-MAS	^{1}H-^{31}P CP-MAS	2D ^{31}P-^{31}P DARR 150 ms	2D ^{31}P-^{31}P DARR 150 ms
Figures	3, 4, 5, S3, S10	3, 4, 5, S7	3, S6, S10	3
ν_r / kHz	17.0	17.0	17.0	17.0
B_0/ T	11.7	11.7	11.7	11.7
transfer I	HP-CP	HP-CP	HP-CP	HP-CP
ν_1(^{1}H)/ kHz	60.0	60.0	60.0	60.0
ν_1(^{31}P)/ kHz	72.3	86.6	72.3	86.6
Shape	Tangent ^{1}H	Tangent ^{1}H	Tangent ^{1}H	Tangent ^{1}H
time / ms	1.0	1.5	1.0	1.5
transfer II	-	-	DARR	DARR
ν_1(^{1}H)/ kHz	-	-	17.0	17.0
time / ms	-	-	150	150
t_1 increments	-	-	320	676
sweep width (t_1) / kHz	-	-	40	40
acquisition time (t_1) / ms	-	-	4.0	8.5
t_2 increments	5120	5120	3072	3072
sweep width (t_2) / kHz	125	125	125	125
acquisition time (t_2) / ms	20.5	20.5	12.3	12.3
^{1}H Spinal64 decoupling power / kHz	90.0	90.0	90.0	90.0
interscan delay / s	3.0	3.0	2.0	2.0
number of scans	4096	4096	384	256
measurement time / h	3.5	3.5	75	104

Table S2 continued.

Sample	primase-DNA^{CT}-ATP^{N}-dGTP^{C}	primase-H145A-DNA^{CT}-ATP^{N}-dGTP^{C}	primase-H145A-DNA^{CT}-ATP^{N}-dGTP^{C}	primase-DNA^{CT}-ATP^{C}-dGTP^{C}
Experiment	2D hNH	2D hNH	2D ^{31}P-^{31}P DARR 50 ms	2D ^{31}P-^{31}P PDSD 200 ms
Figure	6	6	S7	S4
ν_r / kHz	100.0	100.0	17.0	17.0
B_0/ T	20	20	11.7	11.7
transfer I	HN-CP	HN-CP	HP-CP	HP-CP

$\nu_1(^1H)$/ kHz	80.0	80.0	60.0	60.0
$\nu_1(X)$/ kHz	24.5	13	73.9	77.5
Shape	Tangent ^{1}H	Tangent ^{1}H	Tangent ^{1}H	Tangent ^{1}H
^{15}N carrier / ppm	70	100	-	-
time / ms	1.9	1.2	1.5	1.0
transfer II	NH-CP	NH-CP	DARR	^{1}H-^{1}H SD
$\nu_1(^1H)$/ kHz	73.5	80.0	17	-
$\nu_1(X)$/ kHz	24.5	13.0	-	-
^{1}H carrier / ppm	4.8	4.8	-	-
time / ms	1.9	1.5	50	200
t_1 increments	512	512	320	676
sweep width (t_1) / kHz	13.8	15.5	40	40
acquisition time (t_1) / ms	18.6	16.5	4.0	8.5
t_2 increments	3072	3072	3072	3072
sweep width (t_2) / kHz	39.7	39.7	125	125
acquisition time (t_2) / ms	38.7	38.7	12.3	12.3
^{1}H Spinal64 decoupling power / kHz	-	-	90.0	90.0
^{1}H swfTPPM decoupling power / kHz	10	10	-	-
interscan delay / s	1.2	1.2	2.0	2.0
number of scans	32	96	256	160
measurement time / h	6	18	47	67.5

Table S2 continued.

Sample	primase-DNA^{CT}-ATP^C-dGTP^C	primase-DNA^{CT}-ATP^C-dGTP^C	PCP:Mg^{2+} (1:10)
Experiment	^{1}H-^{31}P CP-MAS	Direct pulsed ^{31}P NMR	2D ^{31}P-^{31}P DARR 150 ms
Figure	S3, S4, S8	S8	S6
ν_r / kHz	17.0	17.0	20.0
B_0/ T	11.7	11.7	11.7
transfer I	HP-CP	-	HP-CP
$\nu_1(^1H)$/ kHz	60.0	-	70.0
$\nu_1(^{31}P)$/ kHz	78.9	-	48.8
Shape	Tangent ^{1}H	-	Tangent ^{1}H
time / ms	1.0	-	1.0
transfer II	-	-	DARR
$\nu_1(^1H)$/ kHz	-	-	17.0
time / ms	-	-	150

t_1 increments	-	-	320
sweep width (t_1) / kHz	-	-	40
acquisition time (t_1) / ms	-	-	4
t_2 increments	5120	5120	3072
sweep width (t_2) / kHz	125	125	100
acquisition time (t_2) / ms	20.5	20.5	15.4
^{1}H Spinal64 decoupling power / kHz	90.0	-	90.0
interscan delay / s	3.0	1.5	3.0
number of scans	512	512	56
measurement time / h	0.33	0.25	16

Supplementary references

(1) Beck, K.; Vannini, A.; Cramer, P.; Lipps, G. *Nucleic Acids Res.*, The archaeo-eukaryotic primase of plasmid pRN1 requires a helix bundle domain for faithful primer synthesis, **2010**, *38*, 6707.

(2) Hediger, S.; Meier, B. H.; Kurur, N. D.; Bodenhausen, G.; Ernst, R. R. *Chem. Phys. Lett.*, NMR cross polarization by adiabatic passage through the Hartmann—Hahn condition (APHH), **1994**, *223*, 283.
